# CPT-11 mitigates autoimmune diseases by suppressing effector T cells without affecting long-term anti-tumor immunity

**DOI:** 10.1038/s41420-024-01983-8

**Published:** 2024-05-04

**Authors:** Hantian Liang, Xinzou Fan, Hao Cheng, Xiao Ma, Yutong Sun, Fang Nan, Jingyang Zhou, Panyin Shu, Wei Zhang, Fengqiong Zuo, Hiroko Nakatsukasa, Dunfang Zhang

**Affiliations:** 1 Department of Biotherapy, State Key Laboratory of Biotherapy and Cancer Center, Collaborative Innovation Center of Biotherapy, West China Hospital, Sichuan University, Chengdu, Sichuan 610041 China; 2 Department of Immunology, West China School of Basic Medical Sciences and Forensic Medicine, Sichuan University, Chengdu, Sichuan China; 3 Laboratory of Microbiology and Immunology, Graduate School of Pharmaceutical Sciences, Chiba University, Chiba, 260-8675 Japan

**Keywords:** Psoriasis, Autoimmunity, Preclinical research

## Abstract

The incidence of autoimmune diseases has significantly increased over the past 20 years. Excessive host immunoreactions and disordered immunoregulation are at the core of the pathogenesis of autoimmune diseases. The traditional anti-tumor chemotherapy drug CPT-11 is associated with leukopenia. Considering that CPT-11 induces leukopenia, we believe that it is a promising drug for the control of autoimmune diseases. Here, we show that CPT-11 suppresses T cell proliferation and pro-inflammatory cytokine production in healthy C57BL/6 mice and in complete Freund’s adjuvant-challenged mice. We found that CPT-11 effectively inhibited T cell proliferation and Th1 and Th17 cell differentiation by inhibiting glycolysis in T cells. We also assessed CPT-11 efficacy in treating autoimmune diseases in models of experimental autoimmune encephalomyelitis and psoriasis. Finally, we proved that treatment of autoimmune diseases with CPT-11 did not suppress long-term immune surveillance for cancer. Taken together, these results show that CPT-11 is a promising immunosuppressive drug for autoimmune disease treatment.

## Introduction

The primary functions of immune systems are aimed at protecting hosts from all kinds of pathogens, including viruses, bacteria and parasites. Normally, effector T cells (Teff cells) and regulatory T cells (Treg cells) maintain immunological homeostasis. The immune system can be trained to not attack the host. This protection of the host from its own immune system is termed immunological tolerance [1]. Immunodeficiencies, including primary immunodeficiency disorders [2], underlie an inability to respond to pathogenic antigens, thereby increasing host susceptibility to infections and cancer. Autoimmune diseases (AID) are considered to be imbalances between Teff cells and Treg cells that result in loss of inflammatory control [3]. AID are characterized by tissue and organ damage caused by autoimmunity. Common damage sites include the brain (multiple sclerosis [MS]), gut (inflammatory bowel diseases), skin (psoriasis), lung (lung fibrosis), endocrine system (type 1 diabetes), and multiple organs involved in systemic inflammation. Gene susceptibility, epigenetics, viruses, and a spectrum of environmental factors are closely associated with AID [4, 5]. In particular, TCRαβ^+^ T cells, including CD4^+^ and CD8^+^ T cells, have been proven to be involved in the immunopathogenesis of AID [6–8]. Although no complete cure for AID has been developed, suppression of systemic inflammation by suppressing T cell-mediated immune responses offers the potential to reduce pain and inflammation and overcome this hurdle.

Irinotecan (CPT-11), a topoisomerase-1 inhibitor that suppresses DNA replication and transcription [9], has been widely used to treat a range of cancers, including lung, gastric, ovarian, cervical, and colorectal cancers [10–14]. At high dosages, CPT-11 causes a spectrum of side effects, including gastrointestinal dysfunction and leukopenia, which account for 36.7% and 33.6% of all side effects, respectively, and are considered the primary adverse effects [15, 16]. Although these side effects limit the use of CPT-11 to treat cancer and suggest broad clinical limitations, these side effects may have positive clinical implications for autoimmunity treatment. Considering the pathogenesis of autoimmune diseases and the fact that CPT-11 causes leukopenia, assessing CPT-11’s potential ability to treat autoimmune diseases is worthwhile.

In the present study, we first demonstrated that CPT-11 treatment could suppress T cell-mediated immune responses in vivo, then elucidated the mechanisms by which CPT-11 treatment caused immune suppression. Our studies demonstrate that CPT-11 can effectively inhibit the proliferation and differentiation of effector T cells by inhibiting T cell glycolysis. We also determined CPT-11 efficacy in treating autoimmune diseases in models of experimental autoimmune encephalomyelitis (EAE) and psoriasis. Data from psoriasis and EAE mouse models collectively demonstrate CPT-11 efficacy in autoimmune disease treatment. We also demonstrate that treatment of autoimmune diseases with CPT-11 does not suppress long-term immune surveillance for cancer.

## Results

### CPT-11 reduces immune cell number and suppresses T helper cell (CD4^+^ T cell) cytokine production

**Fig. 1:**
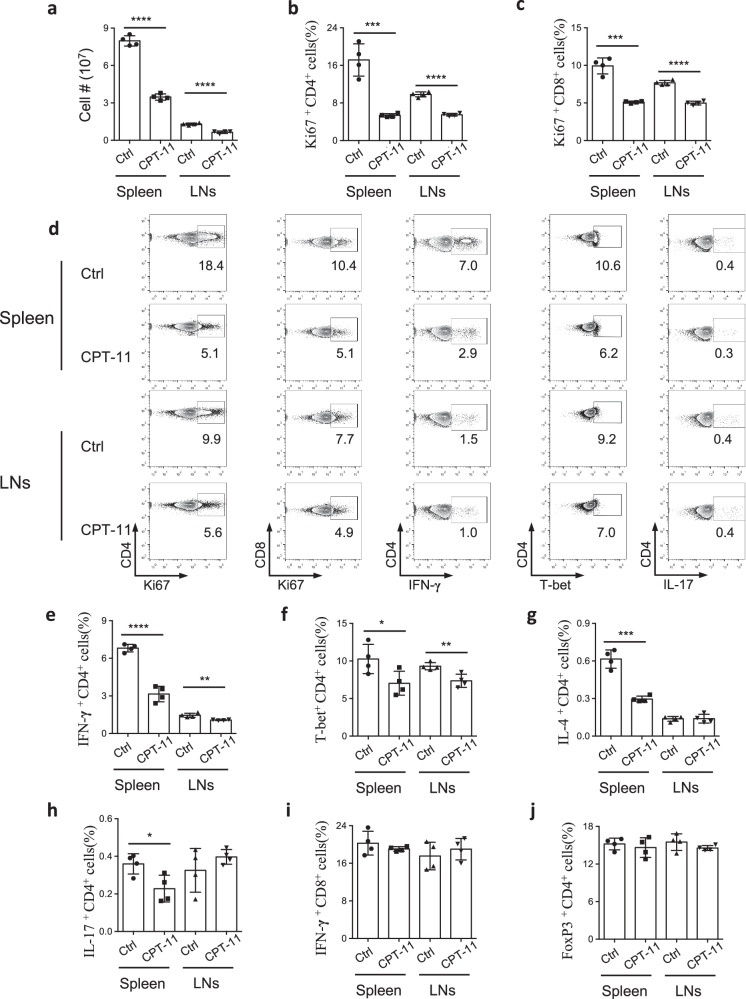
CPT-11 suppresses immune cell proliferation and reduces cytokine production by T cells. C57BL/6 mice were treated with CPT-11, and immune responses were determined by flow cytometry (FCM). **a** Total number of immune cells in spleen and lymph nodes (LNs) of mice treated with PBS (control) or CPT-11 (*n* = 4 mice per group). **b**, **c** Bar graphs show the frequency of Ki67^+^ CD4^+^ and Ki67^+^CD8^+^ T cells in the indicated groups. **d** Representative fluorescence-activated cell sorting (FACS) plots of indicated groups. **e**–**j** Bar graphs showing frequencies of IFN-γ^+^ CD4^+^ T (Th1) cells, T-bet^+^ CD4^+^ T (Th1) cells, IFN-γ^+^ CD8^+^ T cells, IL-17^+^ CD4^+^ T (Th17) cells, IL-4^+^ CD4^+^ T (Th2) cells, and FoxP3^+^ CD4^+^ Treg cells in indicated groups. Data are representative of two independent experiments. Summary data are presented as mean ± s.d. *p < 0.05, **p < 0.01, ***p < 0.001, ****p < 0.0001; by unpaired two-tailed Student’s t-tests. See also Supplementary Fig. 1.

To determine whether CPT-11 could suppress immune responses, we treated C57BL/6 mice with CPT-11. We found that total immune cell numbers were reduced in both the spleen and lymph nodes (LNs) (Fig. 1a). Ki67 antigen is a marker of cellular proliferation [17]. Its expression was markedly decreased in CD4^+^ and CD8^+^ T cells after CPT-11 treatment, indicating that T cell growth was significantly suppressed (Fig. 1b–d). Besides, CPT-11 treatment also caused increased cell apoptosis in both the spleen and LNs (Supplementary Fig. 1a, b), showing that CPT-11 can reduce immune cell numbers by suppressing cell proliferation and promoting cell apoptosis. Expression of the T helper 1 (Th1) cell specific cytokine IFN-γ and the Th1 cell specific transcription Factor T box transcription factor (T-bet) were prominently decreased (Fig. 1d–f). The Th2 cell specific cytokine IL-4, and the Th17 cell specific cytokine IL-17 were suppressed in the spleen (Fig. 1d, g, h, Supplementary Fig. 1c). In contrast, no significant change in the frequency of IFN-γ-producing CD8^+^ cells was observed (Fig. 1i, Supplementary Fig. 1c). In addition, the frequency of Treg cells, key suppressors of inflammation, did not change (Fig. 1j, Supplementary Fig. 1c). Taken together, these data show that CPT-11 inhibits the immune response by reducing the number of immune cells and suppressing CD4^+^ T cell cytokine production.

### CPT-11 suppresses systemic inflammation in response to CFA immune challenge

**Fig. 2:**
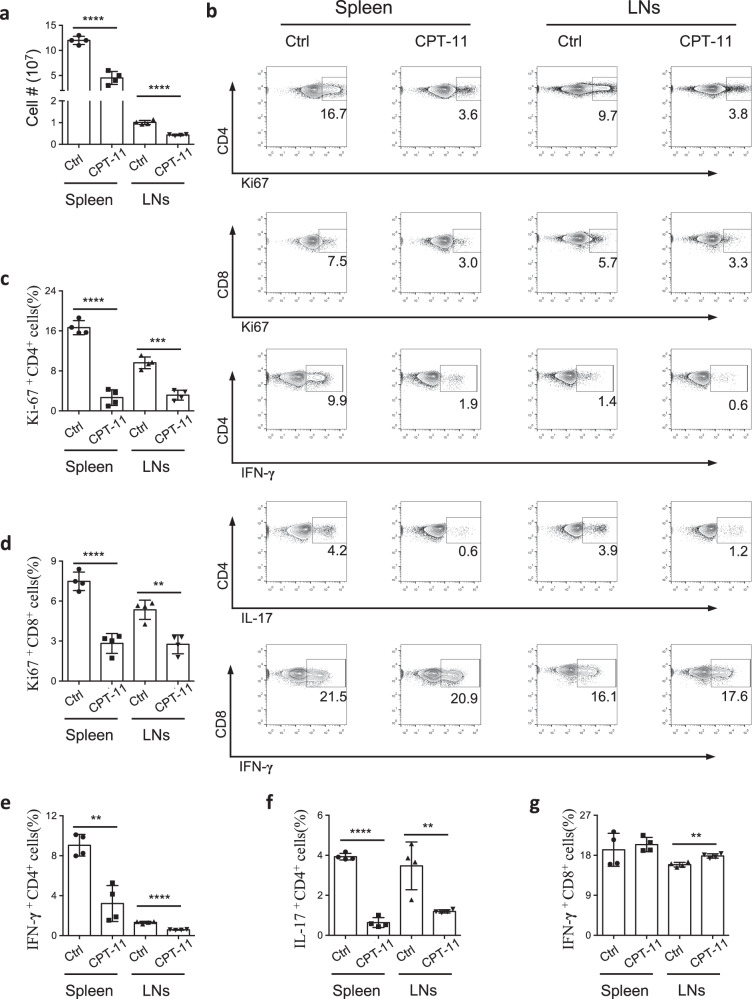
CPT-11 suppresses systemic inflammation under CFA immune challenge. C57BL/6 mice were challenged with CFA (subcutaneous injection) and treated with CPT-11 or PBS, and immune responses in spleen and LNs were determined using FCM. **a** Total number of immune cells in the spleen and LNs of mice treated with PBS (control) or CPT-11. (*n* = 4 mice per group). **b** Representative FACS plots of indicated groups. **c**–**g** Bar graphs showing frequencies of Ki67^+^ CD4^+^ and Ki67^+^CD8^+^ T cells, IFN-γ^+^ CD4^+^ Th1 cells, IL-17^+^ CD4^+^ Th17 cells, and IFN-γ^+^ CD8^+^ cells from indicated mice. Data are representative of two independent experiments. Summary data are presented as mean ± s.d. **p < 0.01, ***p < 0.001, ****p < 0.0001; by unpaired two-tailed Student’s t-tests. See also Supplementary Fig. 2.

To determine whether CPT-11 treatment produces a therapeutic effect under immune challenge, we challenged C57BL/6 mice with CFA and treated them with either PBS or CPT-11. CPT-11 reduced the total number of immune cells (Fig. 2a) and suppressed proliferation of CD4^+^ and CD8^+^ T cells (Fig. 2b–d). Moreover, IFN-γ and IL-17 production, and T-bet expression in CD4^+^ T cells were suppressed significantly after CPT-11 treatment (Fig. 2b, e, f, Supplementary Fig. 2a, b), and the frequency of IL-4-producing Th2 cells was also suppressed (Supplementary Fig. 2a, c), whereas IFN-γ production by CD8^+^ T cells was not suppressed by CPT-11 (Fig. 2b, g). Moreover, Foxp3^+^ Treg cells showed no reduction in their numbers in draining lymph nodes, although Treg cells were decreased in the spleen (Supplementary Fig. 2a, d). Taken together, these data show that CPT-11 suppresses systemic inflammation by reducing immune cell numbers and suppressing CD4^+^ T cell cytokine production under CFA immune challenge.

### CPT-11 suppresses CD4^+^ T cell proliferation, induces CD4^+^ T cell apoptosis, and suppresses CD4^+^ T cell cytokine production in vitro

**Fig. 3:**
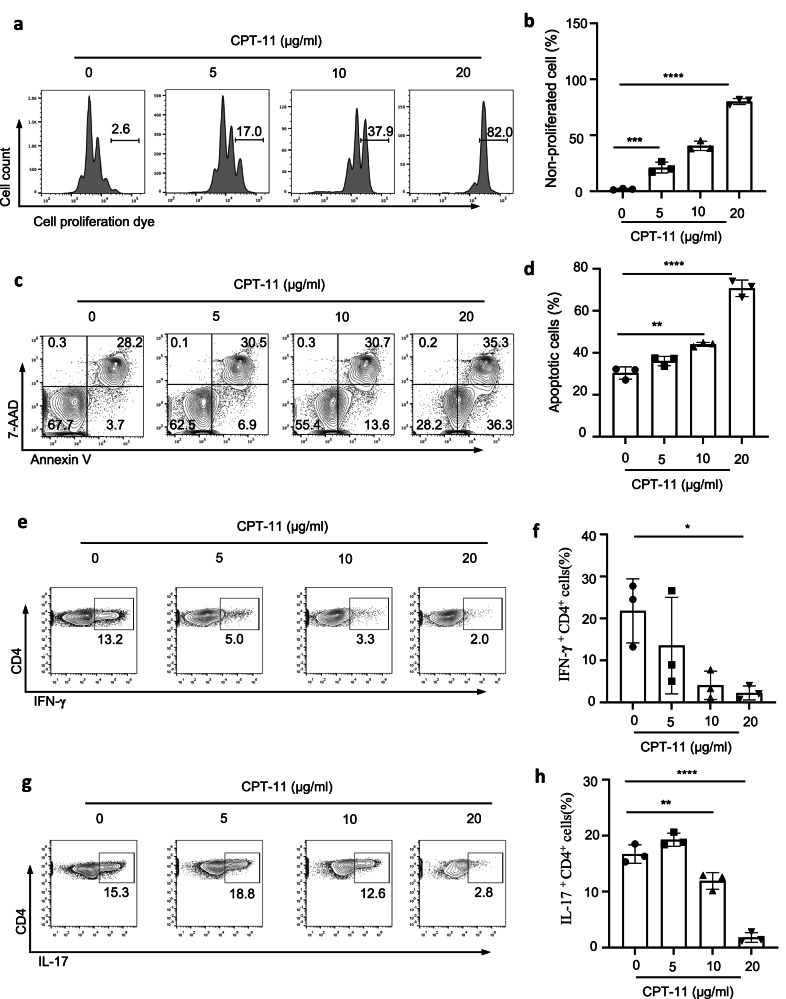
CPT-11 suppresses T cell immune responses in vitro. CD4^+^CD25^−^CD62L^high^ (naive) T cells isolated from spleen and LNs of C57BL/6 mice were cultured with anti-CD3 and anti-CD28, with or without CPT-11 for 1-3 d. Cell proliferation, cell apoptosis, and cell differentiation were determined using FCM (*n* = 3). **a**, **b** Representative FACS plots (**a**) and bar graph (**b**) showing non-proliferative T cell frequencies in T cells cultured for 3 d. **c**, **d** Representative FACS plots (**c**) and bar graph (**d**) showing apoptotic T cell frequencies in T cells cultured for 24 h. **e**, **f** Representative FACS plots (**e**) and bar graph (**f**) showing the frequency of Th1 cells in T cells cultured for 3 d in the presence of IL-12. **g**, **h** Representative FACS plots (**g**) and bar graph (**h**) showing frequencies of Th17 cells among T cells cultured for 3 d in the presence of TGF-β and IL-6. Data are representative of three independent experiments (**a**, **c**, **e**, **g**) or are pooled from three independent experiments (**b**, **d**, **f**, **h**). Summary data are presented as mean ± s.d. *p < 0.05, **p < 0.01, ***p < 0.001, ****p < 0.0001; by unpaired two-tailed Student’s t-tests. See also Supplementary Fig. 3.

Ki67 expression was suppressed in T cells after CPT-11 treatment in vivo (Figs. 1b, d, 2b, c), suggesting that CPT-11 suppressed T cell proliferation. To further investigate how CPT-11 reduces immune cell numbers, we cultured CD4^+^ T cells with different concentrations of CPT-11 in vitro and found that T cell activation was not affected (Supplementary Fig. 3a). However, CD4^+^ T cell proliferation was suppressed (Fig. 3a, b), and CD4^+^ T cell apoptosis was increased (Fig. 3c, d). Considering that IFN-γ- and IL-17-producing CD4^+^ T cell numbers were suppressed in CPT-11-treated mice, we also investigated Th1 and Th17 cell differentiation in CPT-11-treated CD4^+^ T cells in vitro. The in vitro results were consistent with our CPT-11-treated mice results (Fig. 3e–h). These data demonstrate that CPT-11 treatment reduces T cell immunity by inducing cell apoptosis, suppressing cell proliferation, and suppressing Th1 and Th17 cell differentiation.

### CPT-11 suppresses CD4^+^ T cell proliferation and cytokine production by inhibiting glycolysis

**Fig. 4:**
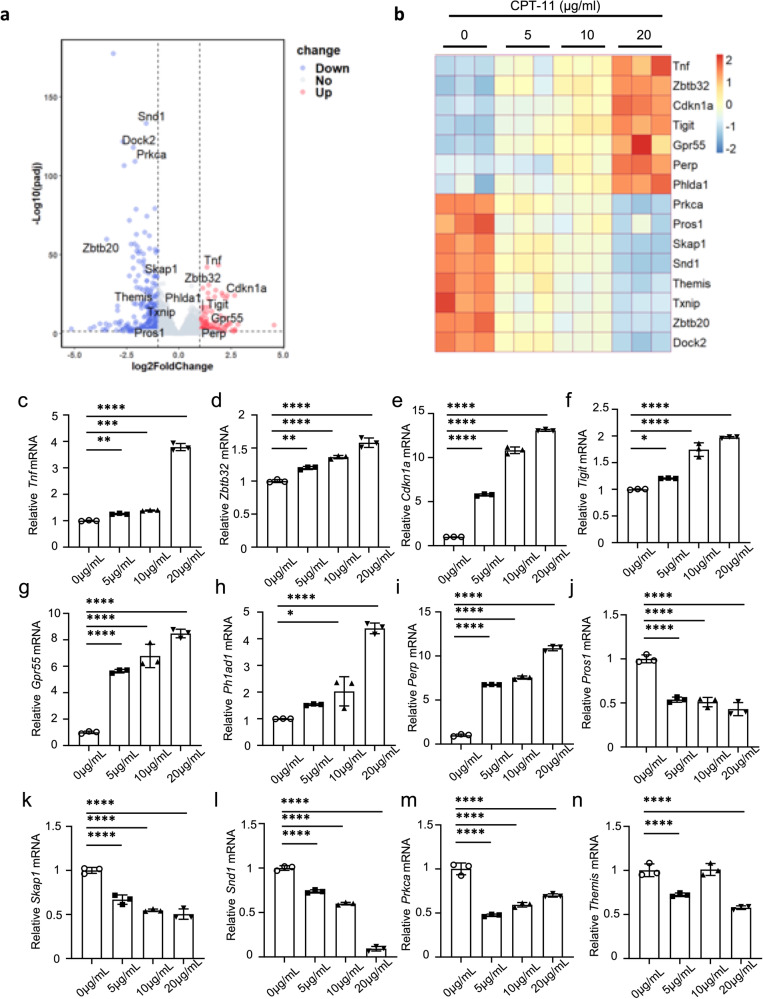
CPT-11 modulates the expression of genes related to CD4^+^ T cell proliferation and apoptosis. Naive T cells isolated from C57BL/6 mice were cultured for 1-3 d with anti-CD3 and anti-CD28 antibodies, with or without CPT-11. **a**, **b** RNA sequencing of T cells cultured for 24 h with 0–20 µg/mL CPT-11. **a** A volcano plot showing differentially expressed genes (DEGs) in T cells treated with 20 μg/mL CTP-11 compared to control sample (*p* < 0.05 and |Log2FoldChange | ≥1). The genes shown in the plot represent apoptosis-related genes. **b** A heat map showing expression levels of apoptosis-related genes (rows; Z normalized per row) that are differentially expressed in T cells cultured with different concentrations (0 µg/mL, 5 µg/mL, 10 µg/mL, and 20 µg/mL) of CPT-11. Darker red color indicates higher relative expression, and darker blue color indicates lower relative expression. **c**–**l** mRNA expression of Tnf, Zbtb32, Cdkn1a, Tigit, Gpr55, Ph1ad1, Perp, Pros1, Skap1 and Snd1 in T cells cultured for 3 d with different concentrations (0 µg/mL, 5 µg/mL, 10 µg/mL, and 20 µg/mL) of CPT-11. **m**, **n** mRNA expression of Prkca and Themis in T cells cultured for 1 d with different concentrations (0 µg/mL, 5 µg/mL, 10 µg/mL, and 20 µg/mL) of CPT-11. Data are pooled from three independent experiments. Summary data are presented as mean ± s.d. *p < 0.05, **p < 0.01, ***p < 0.001, ****p < 0.0001; by one-way analysis of variance (ANOVA) with Tukey’s post hoc test. See also Supplementary Fig. 4.

To further investigate the mechanisms by which CPT-11 induces immune suppression of T cells, we analyzed the transcriptomes of naïve CD4^+^ T cells treated with different concentrations of CPT-11 in the presence of TCR stimulation in vitro. Volcano plot and heat map revealed differential genes related to T cell proliferation and apoptosis in CPT-11-treated CD4^+^ T cells (Fig. 4a, b). Expression of Cdkn1a, Perp, Phlda1, and Tnf was upregulated in CPT-11-treated T cells (Fig. 4a, b), and the proteins encoded by these genes have been shown to induce T cell apoptosis [18–21]. Moreover, expression of Gpr55, Tigit, and Zbtb32 was upregulated in CPT-11-treated T cells (Fig. 4a, b), and the proteins encoded by these genes have been shown to suppress T cell proliferation [22–25]. In contrast, transcript levels of Dock2, Prkca, Pros1, Skap1, Themis, and Zbtb20 were down-regulated in CPT-11-treated T cells (Fig. 4a, b), and the proteins encoded by these genes have been shown to promote cell proliferation [26–32]. Interestingly, studies have suggested that SND1 and TXNIP play important roles in Th1 or Th17 cell-mediated immune responses [33, 34]. These two genes were also down-regulated in CPT-11-treated CD4^+^ T cells (Fig. 4a, b). To confirm the changes of the RNA-seq analysis, mRNA levels of genes associated with T cell proliferation, apoptosis and function were also measured and validated by quantitative RT-PCR (Fig. 4c–n, Supplementary Fig. 4a–c). These data show that CPT-11 modulates the expression of genes that influence T cell proliferation and apoptosis, thereby inhibiting T cell-mediated immunity.

**Fig. 5:**
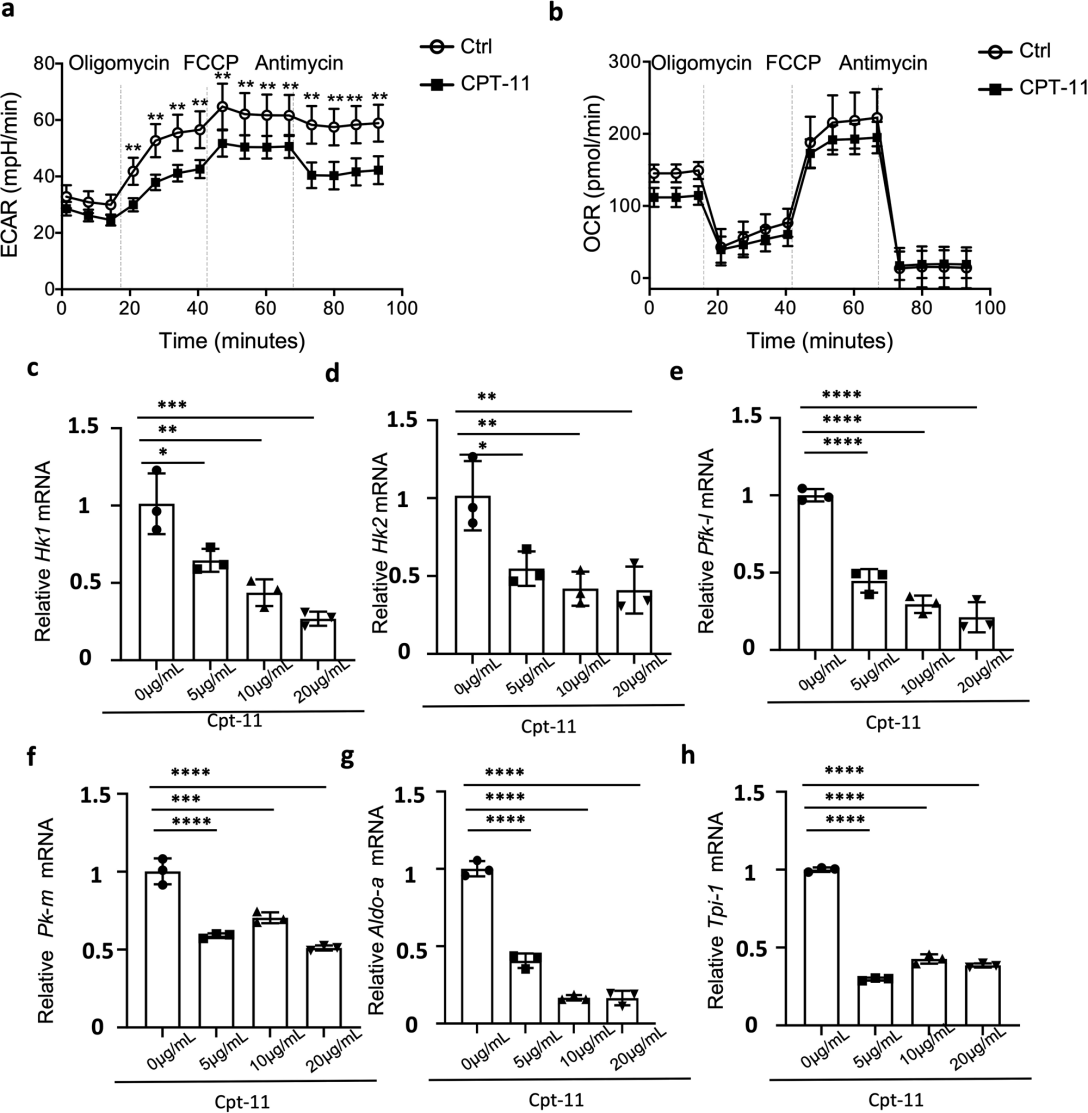
CPT-11 suppresses CD4^+^ T cell proliferation and cytokine production by inhibiting glycolysis. Naive T cells isolated from C57BL/6 mice were cultured for 3 d with anti-CD3 and anti-CD28 antibodies, with or without CPT-11. **a** Extracellular Acidification Rate (ECAR) of T cells cultured with or without CPT-11 in basal state and after addition of Oligomycin, FCCP, and Antimycin. **b** Oxygen Consumption Rate (OCR) of T cells cultured with or without CPT-11, at basal state, and after addition of Oligomycin, FCCP, and Antimycin. **c**–**h** mRNA expression of Hk1, Hk2, Pfk-L, Pk-M, Aldo-a and Tpi-1 in T cells cultured with different concentrations (0 µg/mL, 5 µg/mL, 10 µg/mL, and 20 µg/mL) of CPT-11. Naive T cells isolated from C57BL/6 mice were cultured for 3 d with anti-CD3 and anti-CD28, with or without 2-DG. Data are representative of three independent experiments (**a**, **b**) or are pooled from three (**c**–**h**) independent experiments. Summary data are presented as means ± s.d. *p < 0.05, **p < 0.01, ***p < 0.001, ****p < 0.0001; by unpaired two-tailed Student’s t-tests (**a**, **b**) or one-way analysis of variance (ANOVA) with Tukey’s post hoc test (**c**–**h**). See also Supplementary Fig. 5.

Since CD4^+^ T cell glycolysis has been shown to be important for T cell proliferation and cytokine production, but not for cell activation [35], we next investigated whether CPT-11 treatment could inhibit CD4^+^ T cell glycolysis. Interestingly, T cell glycolysis was significantly suppressed, as CPT-11-treated CD4^+^ T cells displayed a markedly reduced acidification rate (Fig. 5a). In contrast, the oxygen consumption rate (OCR) of CPT-11-treated CD4^+^ T cells showed no significant change (Fig. 5b), suggesting that CPT-11 does not affect oxidative phosphorylation or fatty acid oxidation. It has been well known that three key glycolysis rate-limiting enzymes, including hexokinase (HK), phosphofructokinase (PFK) and pyruvate kinase (PK), play crucial roles in regulating three irreversible stages. The hexokinases, the first enzymes were dedicated to glycolysis, catalyzed the conversion from glucose to glucose-6-phosphate. Whilst there were four distinct isozymes of hexokinase, the mainly two isoforms, HK1 and HK2, were expressed by T cells [36, 37]. In addition, PFK and PK family all contains various isoforms. There is ample evidence that PFK-L and PK-M is the dominant isoform in T cells [38, 39]. To investigate the mechanism by which glycolysis is suppressed, we measured the mRNA expression of HK1, HK2, PFK-L, PK-M, Aldo-a and Tpi-1, and found that the expression of all these genes was suppressed after CPT-11 treatment (Fig. 5c–h). These data demonstrate that the inhibition of glycolytic enzymes by CPT-11 is broad-spectrum. To further confirm that CPT-11 induced inhibition of glycolysis can limit T cell immunity, we cultured CD4^+^ T cells with 2-DG, an inhibitor of Hk1 and Hk2. We found that T cell proliferation and IFN-γ production were both suppressed in the presence of 2-DG (Supplementary Fig. 5a–d). Taken together, CPT-11 suppressed CD4^+^ T-cell proliferation and cytokine production by reducing T-cell glycolysis.

### CPT-11 suppresses progression of psoriasis

**Fig. 6:**
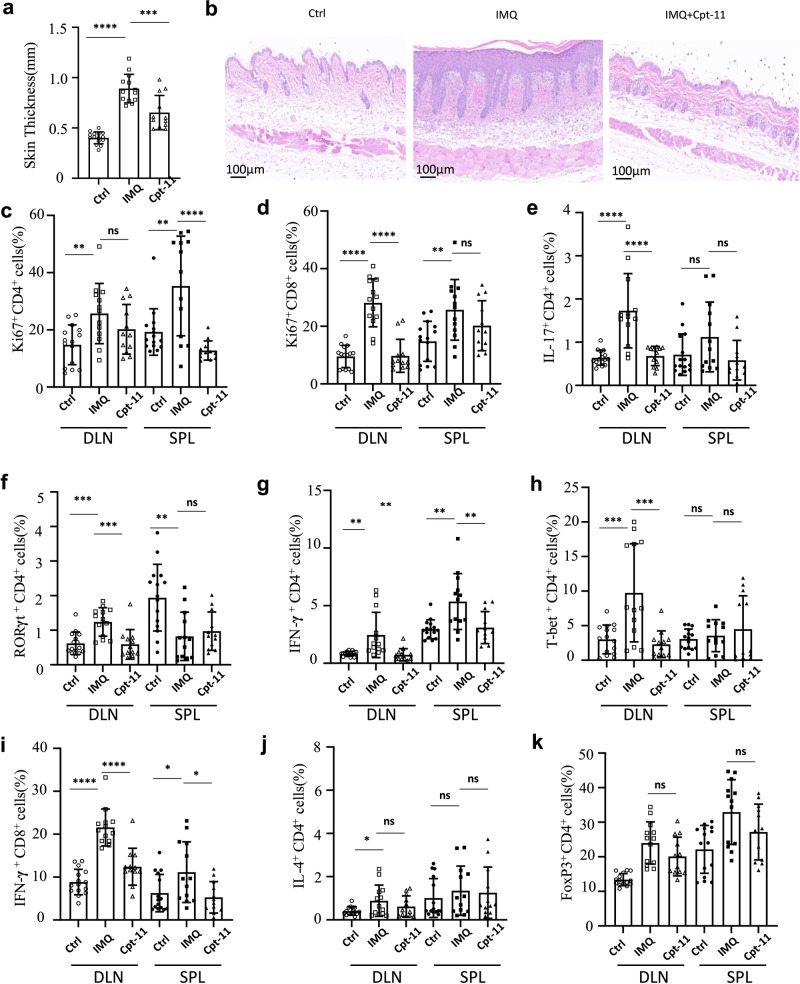
CPT-11 suppresses progression of psoriasis. C57BL/6 mice were administered IMQ cream on a 2.5 cm × 2.5 cm patch of shaved back skin daily for 7 consecutive days, and were injected with CPT-11 or PBS intraperitoneally once per day (*n* = 12 mice per group). **a** Statistical analysis of epidermal thickness. **b** Representative histological skin images. **c**–**k** Bar graphs showing frequencies of Ki67^+^ CD4^+^ T cells (**c**), Ki67^+^CD8^+^ T cells (**d**), IL-17^+^ CD4^+^ Th17 cells (**e**), RORγt^+^ CD4^+^ Th17 cells (**f**), IFN-γ^+^ CD4^+^ Th1 cells (**g**), T-bet^+^ CD4^+^ Th1 cells (**h**), IFN-γ^+^ CD8^+^ T cells (**i**), IL-4^+^ CD4^+^ Th2 cells (**j**) and FoxP3^+^ CD4^+^ Treg cells (**k**) in the spleen (SPL) and draining lymph nodes (DLN) of indicated groups. Data are representative of three independent experiments (**a**, **b**) or are pooled from three independent experiments (**c**–**k**). Summary data are presented as mean ± s.d. *p < 0.05, **p < 0.01, ***p < 0.001, ****p < 0.0001; by a one-way analysis of variance (ANOVA) with Tukey’s post hoc test. See also Supplementary Fig. 6.

Since CPT-11 can reduce T-cell numbers and suppress T-cell-mediated inflammation, we investigated whether it could be used to treat autoimmunity. We established an experimental mouse model of classical IMQ-induced psoriasis [40] to determine whether CPT-11 could suppress psoriasis. Skin thickness and histological analysis demonstrated that the severity of psoriasis was significantly alleviated in CPT-11-treated mice compared to that in PBS-treated mice (Fig. 6a, b). By investigating the immune responses in these mice using flow cytometry (FCM), we found that expression of Ki67, a protein that is strictly associated with cell proliferation and viewed as a proliferation marker [17], was suppressed in CD4^+^ and CD8^+^ cells in mice treated with CPT-11 (Fig. 6c, d, Supplementary Fig. 6a, b). Simultaneously, expression of IL-17 and retinoic acid-related orphan receptor gamma t (RORγt, the key transcription factor of Th17 cells) were markedly down-regulated (Fig. 6e, f, Supplementary Fig. 6c–d). Consistent with these findings, expression of IFN-γ and T-bet, the key transcription factor of Th1 cells, were significantly decreased (Fig. 6g, h, Supplementary Fig. 6e, f). The frequency of IFN-γ-producing CD8^+^ T cells was also reduced (Fig. 6i, Supplementary Fig. 6g). In contrast, the frequencies of Th2 and Foxp3^+^ Treg cells did not change in CPT-11-treated mice (Fig. 6j, k, Supplementary Fig. 6h, i). These data show that the immune responses of Th17 cells, Th1 cells, and IFN-γ-producing CD8^+^ T cells experienced a marked decline in mice with psoriasis treated with CPT-11. Overall, the immune responses in CPT-11-treated psoriasis mice were similar to those of healthy control mice (Fig. 6). Taken together, CPT-11 treatment suppressed psoriasis in a mouse disease model by suppressing T cell proliferation and Th1 and Th17 cell differentiation.

### CPT-11 inhibits development of EAE

**Fig. 7:**
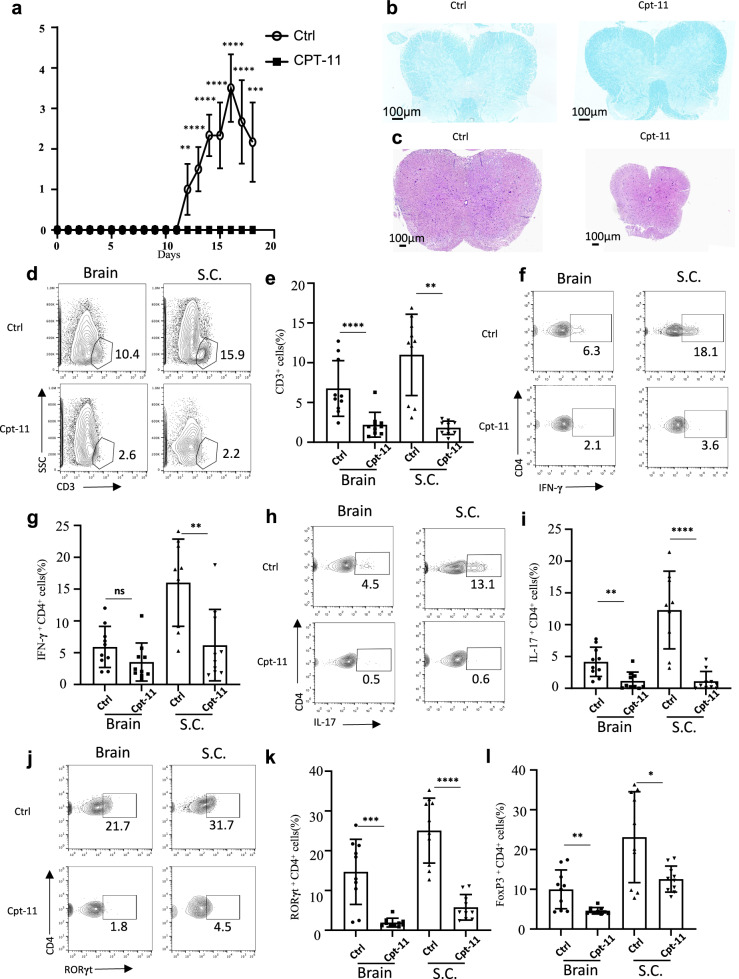
CPT-11 inhibits development of EAE. C57BL/6 mice were subcutaneously immunized with MOG peptide 35–55 emulsified in complete Freund’s adjuvant to induce EAE, and treated with CPT-11 or PBS daily from day 9. **a** EAE clinical scores of the indicated groups (*n* = 10 mice per group). **b** Representative Luxol Fast Blue (LFB) staining of cervical spinal cord sections. **c** Representative histological images of cervical spinal cord sections. **d**, **e** Representative FACS plots (**d**) and bar graph (**e**) showing frequencies of CD3^+^ T cells in the brain and spinal cord. **f**, **g** Representative FACS plots (**f**) and bar graph (**g**) showing frequencies of IFN-γ^+^ CD4^+^ Th1 cells in brain and spinal cord. **h**–**k** Representative FACS plots (**h**, **j**) and bar graphs (**l**, **k**) showing frequencies of Th17 cells in brain and spinal cord. **l** Bar graph showing frequencies of Foxp3^+^Treg cells in brain and spinal cord. Data are representative of two independent experiments (**a**–**c**) or are pooled from two independent experiments (**d**–**l**). Summary data are presented as mean ± s.d. *p < 0.05, **p < 0.01, ***p < 0.001, ****p < 0.0001; by unpaired two-tailed Student’s t-tests. See also Supplementary Fig. 7.

CPT-11 can cross the blood–brain barrier (BBB), although its concentration in the central nervous system (CNS) is lower than that in peripheral blood [41, 42]. Therefore, whether CPT-11 can suppress CNS inflammation in conditions such as MS is worth determining. To investigate this, we established an EAE model, as an experimental mouse model of MS. C57BL/6 mice were immunized with myelin oligodendrocyte glycoprotein (MOG) peptide 35–55 in complete Freund’s adjuvant (CFA) to induce EAE [43] and were treated with CPT-11 or PBS on day 9. The development of EAE was completely suppressed in mice treated with CPT-11 (Fig. 7a). Luxol fast blue (LFB) staining showed that demyelination of the cervical spinal cord was also completely suppressed in mice treated with CPT-11 (Fig. 7b). Histological analysis and immune cell counting results also showed that EAE mice treated with CPT-11 had much reduced infiltration of inflammatory cells into the CNS compared to EAE mice treated with PBS (Fig. 7c, Supplementary Fig. 7a). In particular, the number of CD3^+^ T cells was significantly reduced in the brain and spinal cord in mice treated with CPT-11 (Fig. 7d, e). Moreover, the frequencies of Th1 (Fig. 7f, g) and Th17 (Fig. 7h–k) cells were reduced in the brain and spinal cord (S.C.) of EAE mice treated with CPT-11, whereas the proportion of Th2 cells did not change significantly (Supplementary Fig. 7b, c). The proportion of Treg cells was significantly reduced (Fig. 7l, Supplementary Fig. 7d), demonstrating that inhibition of EAE by CPT-11 does not depend on Treg cell induction or recruitment. Collectively, our data suggest that CPT-11 could treat EAE by suppressing effector T cells and inhibiting Th1 and Th17 cells.

### CPT-11 therapy of autoimmune diseases does not affect long-term anti-tumor immunity

**Fig. 8:**
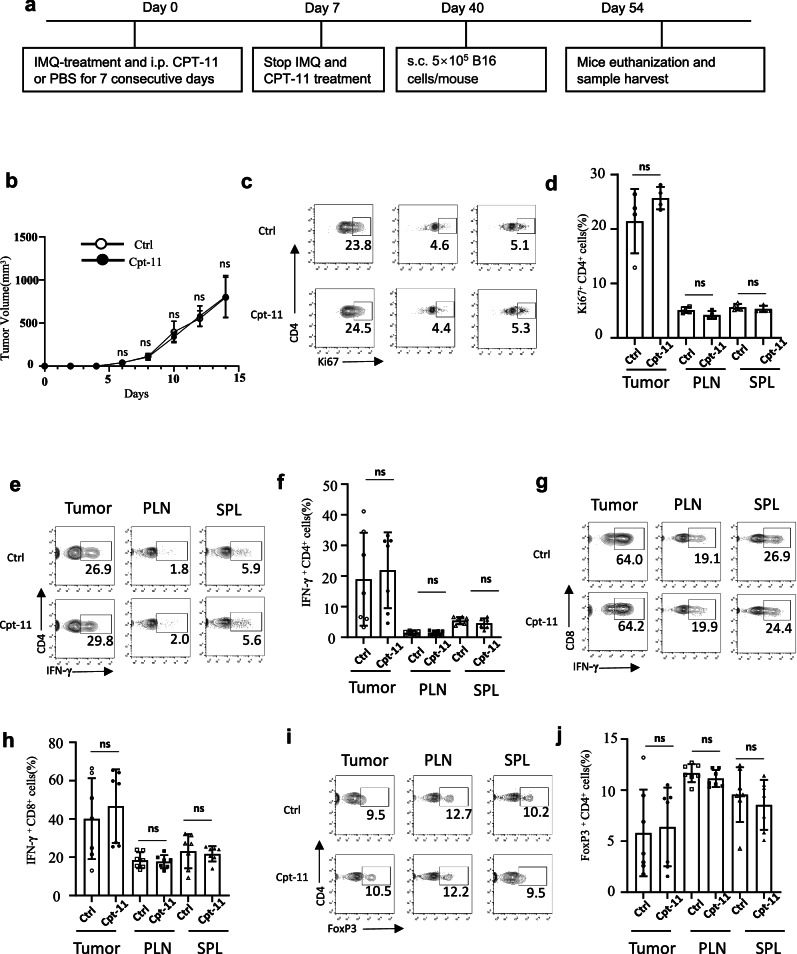
CPT-11 therapy of autoimmune diseases does not affect long-term anti-tumor immunity. C57BL/6 mice were administered IMQ cream on shaved 2.5 cm × 2.5 cm patches of back skin daily for 7 consecutive days and were injected with CPT-11 or PBS intraperitoneally once per day. Approximately 5 weeks after psoriasis induction and treatment, the mice were injected with B16 cells to establish a tumor-bearing model (*n* = 7 mice per group). **a** Experimental scheme of the B16 tumor-bearing model after psoriasis induction and treatment. **b** Tumor growth curves. **c**–**j** Representative FACS plots (**c**, **e**, **g**, **i**) and Bar graphs (**d**, **f**, **h**, **j**) showing frequencies of Ki67^+^ CD4^+^ T cells (**c**, **d**), IFN-γ^+^ CD4^+^ Th1 cells (**e**, **f**), IFN-γ^+^ CD8^+^ cells (**g**, **h**), and FoxP3^+^ CD4^+^ Treg cells (**i**, **j**). Data are representative of two independent experiments (**b**–**d**) or are pooled from two independent experiments (**e**–**j**). Summary data are presented as mean ± s.d. *p < 0.05, **p < 0.01, ****p < 0.0001; by unpaired two-tailed Student’s t-tests. See also Supplementary Fig. 8.

Our animal studies on psoriasis and EAE showed that CPT-11 can effectively treat autoimmune diseases. In the meanwhile, we also found that mice treated with CPT-11 experienced a decrease in body weight in psoriasis model (Supplementary Fig. 8a), showing a possible side effect of CPT-11 treatment. Therefore, an important question to confirm is whether systemic immunosuppression by CPT-11 will compromise protective immune responses such as anti-tumor immunity. To investigate whether CPT-11 treatment affects anti-tumor immune responses, we established a B16 melanoma tumor-bearing model in mice with psoriasis. These mice were treated with CPT-11 or PBS, and approximately 5 weeks later, B16 cells were subcutaneously injected to assess tumor growth (Fig. 8a). Tumor growth was similar in psoriatic-rehabilitated mice treated with CPT-11 or PBS (Fig. 8b) and T cell proliferation was comparable in mice treated with CPT-11 or PBS (Fig. 8c, d). Frequencies of Th1 cells, IFN-γ^+^ CD8^+^ T cells, Foxp3^+^ Treg cells, Th17 cells, and Th2 cells were virtually identical in the spleen, peripheral lymph nodes (PLN), and tumors of mice treated with CPT-11 or PBS (Fig. 8e–j, Supplementary Fig. 8b–e). These data show that anti-tumor immune responses and systemic immune responses were similar in CPT-11-treated and control mice. Therefore, CPT-11 therapy can effectively suppress autoimmune diseases, and does not exert a long-term effect on protective anti-tumor immune responses.

## Discussion

Over the past few decades, the incidence of autoimmune diseases has dramatically increased. This increase is positively correlated with economic development [44–46]. Although many immunotherapeutic strategies have been developed in preclinical and clinical studies, immunosuppressive drug therapy remains the mainstream clinical treatment [47–49]. A range of autoimmune diseases result from poorly-defined interactions between environmental and internal cues. The cause that difficult development of targeted treatments ascribed to complex immune cells interacting networks and “unknown trigger factors”. Immunosuppressive drugs play a crucial role in alleviating symptoms and preventing further damage by suppressing immune system’s function. There is mounting immunosuppressive drugs in the treatment of aberrant immune disorders, including antibodies, calcineurine inhibitors, mammalian target of rapamycin inhibitors (mTORi), and steroid hormones. Basiliximab, a chimeric monoclonal antibody, binds to interleukin-2 receptor alpha chain (CD25) and blocks this signaling transduction to suppress the expansion of T cells [50]. Alemtuzumab is a depleting monoclonal antibody directed against the CD52 and used to induce the depletion of T and B lymphocytes [51, 52]. The significant economic burden has proved challenging with regard to the application of antibodies. Calcineurin inhibitors, such as cyclosporine A, impair T cells activation and proliferation through the inhibition of the translocation of activated nuclear T-cell transcription factor (NF-AT), which inhibits IL-2 gene transcription [53]. However, some studies have been reported that a series of side effects are hypertension, dyslipidemia, Multiple electrolyte derangements and major in hepatotoxic [54, 55]. mTORi suppress the proliferation and differentiation of T and B lymphocytes, but the increased mortality in SLE patients caused by increased risk of infection, is a significant issue [56, 57]. Steroid hormones can regulate the development and function of immune cells to confer it potent immune-suppressive and anti-inflammatory function. Althought steroid hormones were considered as wonder drugs to ameliorate various inflammatory diseases, their long-term use can bring many side effects, and it may have the opposite effect on B cells [58]. Moreover, the responses of patients with autoimmune diseases to immunosuppressive drugs vary significantly, and even in patients who experience good efficacy, it is difficult to achieve lifelong remission of the disease. Therefore, the development of new immunosuppressive drugs remains an important objective [59, 60]. To determine whether CPT-11 holds promise for autoimmune disease treatment, we investigated the effects of CPT-11 treatment in mouse models of EAE and psoriasis. Changes in immune responses after CPT-11 treatment were determined in healthy C57BL/6 and CFA-challenged mice. In both models, T cell immune responses were significantly suppressed. We discovered that CPT-11 alleviates autoimmunity in both EAE and psoriasis models. T cell-mediated immune responses were also investigated after CPT-11 treatment of EAE and psoriasis mice. We found that T cell numbers and T cell cytokine production were both suppressed. In particular, T cell number and cytokine production by Th1 and Th17 cells were significantly inhibited after CPT-11 treatment in all mouse models, whereas Th2 cells and Treg cells did not change significantly. These findings indicate that CPT-11 is effective in autoimmune treatment and mainly suppresses Th1 and Th17 immune responses. These findings reveal a previously unrecognized drug candidate for the treatment of autoimmune diseases.

To elucidate the mechanism by which CPT-11 suppressed pro-inflammatory effector T cells, naïve T cells were isolated and cultured with CPT-11 in vitro. Consistent with our in vivo results, CPT-11 inhibited T cell proliferation, promoted T cell apoptosis, and inhibited Th1 and Th17 cell differentiation. Live cell metabolism analysis revealed that CPT-11 significantly inhibited glycolysis in T cells. This conclusion is also supported by the decrease in HK1 and HK2 (key enzymes involved in glycolysis) mRNA expression after CPT-11 treatment. In addition, when T cells were treated with the glycolysis inhibitor 2-DG, a phenotype consistent with that of CPT-11 appeared, proving that CPT-11 suppresses the immune response of effector T cells by inhibiting glycolysis. Because we were unable to obtain naïve human T cells for this study, the regulatory function of CPT-11 on human T cell proliferation and differentiation was not studied. Future studies on CPT-11 in autoimmune disease treatment should focus on its effects on human T cells. Nevertheless, as it is well accepted that targeting effector T cell immune responses or T cell glycolysis is a promising strategy to treat autoimmunity [61, 62], our study demonstrates that CPT-11 is an effective drug for treating autoimmune diseases by suppressing T cell-related immune responses.

Because immunosuppressive drugs can cause systemic immune suppression, patients taking long-term immunosuppressive drugs to treat autoimmune diseases may be more susceptible to infections and tumors. To investigate whether CPT-11 treatment causes long-term suppression of protective immune responses, we established a B16 tumor-bearing model in mice with psoriasis treated with CPT-11 to evaluate the strength of their anti-tumor immune responses. Encouragingly, CPT-11 treatment of mice with psoriasis did not dampen anti-tumor immunity, indicating that CPT-11 treatment did not suppress long-term protective immune responses. Maintaining immune surveillance of cancer while effectively suppressing autoimmune diseases has long been a difficult challenge [63]. Our research results suggest that while CPT-11 effectively suppresses the immune response in autoimmune diseases, it does not seriously inhibit the later anti-tumor immune response, showing great potential for the safe treatment of autoimmune diseases.

Previous studies have shown that CPT-11 causes a series of significant side effects, including gastrointestinal dysfunction [64]. In the current study, we also found that mice treated with CPT-11 experienced a decrease in body weight in psoriasis model. We have two viable avenues for addressing negative side effects. We can control their severity through dietary intervention, physical therapy, and drug intervention to achieve clinical treatment effects [65], or we can adjust the dosage of CPT-11 to minimize side effects while ensuring efficacy [66]. Some significant gaps can be present in the transition from preclinical to clinical dosing schedules. During the aforementioned treatment process, the CPT-11 dose was 50 mg/kg body weight. This suggests that future studies should discuss strategies for dose optimization and highlight strategies for integrating dose optimization into pre-marketing drug development.

In summary, our study has demonstrated that CPT-11 can effectively inhibit the proliferation and differentiation of effector T cells by inhibiting T cell glycolysis. Data from psoriasis and EAE mouse models collectively demonstrate the efficacy of CPT-11 for treating autoimmune diseases, and our results indicate that the treatment of autoimmune diseases with CPT-11 does not suppress long-term immune surveillance for cancer.

## Materials and methods

### Antibodies and reagents

The following chemicals were purchased from the indicated manufacturers: purified anti-mouse CD3 (145–2C11, Bio X Cell, # BE0001–1), purified anti-mouse CD28 (37.51, Bio X Cell, # BE0015–1), recombinant mouse IL-12 (R&D Systems, #419-ML-500), Freund’s adjuvant, incomplete (IFA) (BD/Difco Laboratories, # 263910), *Mycobacterium tuberculosis* (BD Biosciences, #231141), DNase I (Millipore Sigma, # DN25), Collagenase IV (Thermo Fisher Scientific, # # 17104–019), PMA (Millipore Sigma, #P8139), Ionomycin calcium salt (Millipore Sigma, #13909), Golgi-Plug Protein Transport Inhibitor (BD Biosciences, #555029), CD4^+^ CD62L^+^ T cell Isolation Kit, mouse (Miltenyi Biotec, #130–106-643), Foxp3/Transcription Factor Staining Buffer Set (Thermo Fisher Scientific, #00–5523-00), Cytofix/Cytoperm Fixation/ Permeabilization Solution Kit (BD Biosciences, #554714), CellTrace™ CFSE Cell Proliferation Kit (Thermo Fisher Scientific, # C34554), Seahorse XF Cell Mito Stress Test Kit (Agilent, # 103015–100), fluorochrome-conjugated antibodies (zombie yellow [Biolegend, #423104]), anti-mouse CD45 (30-f11, Thermo Fisher Scientific, #56-0451-82), anti-mouse TCRβ (H57-597, Thermo Fisher Scientific, # 47–5961-82), anti-mouse CD4 (RM4-5, Thermo Fisher Scientific, #45-0042-82), anti-mouse CD8α (53-6.7, Thermo Fisher Scientific, #11-0081-85), anti-mouse CD8β (H35-17.2, Thermo Fisher Scientific, 11-008S-85), anti-mouse CD62L (MEL-14, Thermo Fisher Scientific, #11-0621-85), anti-mouse CD44 (IM7, Thermo Fisher Scientific, #17-0441-83), anti-mouse CD25 (PC61.5- Thermo Fisher Scientific, #45-0251-82), anti-mouse CD69 (H1.2F3, Thermo Fisher Scientific, #12-0691-83), anti-mouse IFN-γ (XMG1.2, Thermo Fisher Scientific, #48-7S11-82), anti-mouse IL-17(eBio17B7, Thermo Fisher Scientific, #25-7177-82), anti-mouse IL-4 (11B11, Thermo Fisher Scientific, #25-7041-82), anti-mouse Ki67 (SolA15, Thermo Fisher Scientific, #25-5698-82), anti-mouse RORγt (B2D, Thermo Fisher Scientific, #17-6981-82), anti-mouse T-bet (eBio4B10, Thermo Fisher Scientific, #12-5825-82), anti-mouse FoxP3 (FJK-16s, Thermo Fisher Scientific, #48-577S-82), 7-AAD Viability Staining Solution (Thermo Fisher Scientific, #00-6993-50), and Annexin V (Thermo Fisher Scientific, #BMS306PE-100), anti-mouse CD16/32 (Biolegend, #101302).

### Experimental model details

Mice C57BL/6 mice were purchased from Shanghai Model Organisms Center, Inc., and bred in our facility under specific pathogen-free conditions. All mice used in these experiments were aged 6–8 weeks. All animal studies were performed in accordance with the guidelines of the Animal Care and Use Committee of West China Hospital, Sichuan University, and were approved by the Animal Care and Use Committees of West China Hospital, Sichuan University. Mice were allocated randomly to treatment groups and ctrl groups in an arbitrary order and assigned a specific number before data collection, However, we didn’t use formal randomization techniques. The data collection and analysis were blindly. No statistical calculation was performed to choose sample size. Sample size was determined by previous similar studies [67–71], and to ensure adequate data values to conduct standard statistical tests.

### CPT-11 treatment of C57BL/6 mice and CFA-challenged mice

To investigate whether CPT-11 could suppress immune responses in healthy mice, eight-week-old male C57BL/6 mice were treated with CPT-11 (50 mg/kg body weight) or PBS daily for 7 consecutive days. To investigate whether CPT-11 could suppress immune responses under immune challenge, eight-week-old male C57BL/6 mice were immunized subcutaneously with complete Freund’s adjuvant, which contained 300 μg/mouse heat-killed *M. tuberculosis* H37Ra, on two different hind flank sites, and treated with 50 mg/kg body weight CPT-11 or PBS daily from day 8 for 7 consecutive days. On the next day after CPT-11 treatment, mice were euthanized and samples were harvested for subsequent experiments.

#### EAE induction and scoring

The EAE model was established as previously described [45, 72]. MOG35–55 peptide (200 μg/mouse) was drawn up into syringes with complete Freund’s adjuvant (which contained 300 μg/mouse heat-killed *M. tuberculosis* H37Ra) to produce an emulsion. Eight-week-old male or female C57BL/6 mice were then immunized subcutaneously with the emulsion on day 0 and treated with 50 mg/kg body weight CPT-11 or PBS daily from day 9 for 7 consecutive days. On the day of immunization and 2 days later, 200 ng/mouse pertussis toxin was administered intraperitoneally. On day 18, mice were euthanized and tissue samples were harvested for subsequent experiments. Mice were observed daily to assess disease development. The EAE clinical scores were as follows: 0, no disease; 1, limp tail or hind limb weakness; 2, limp tail and hind limb weakness; 3, hind limb paralysis; 4, hind limb paralysis and forelimb weakness; and 5, moribund.

#### Psoriasis

This model was used as previously described [73, 74]. Eight-week-old female C57BL/6 mice were treated with 60 ~ 65 mg commercially available 5% IMQ cream (Med-shine Pharma, Chengdu, China) on shaved 2.5 cm × 2.5 cm back skin daily for 7 consecutive days, and treated with 50 mg/kg body weight CPT-11 or PBS daily. On day 7, mice were euthanized and samples were harvested for subsequent experiments.

### Method Details

#### Cell isolation from brain and spinal cord

To obtain lymphocytes from brain and spinal cord, as previously reported [75], tissues from EAE model mice were cut into small pieces and resuspended in PBS containing collagenase IV (4 mg/mL) and DNase (2 mg/mL) for 30–40 min. Undigested tissues were then mashed through a 70 μm cell strainer to yield cell suspensions. Spleens and lymph nodes were directly passed through a 70 μm cell strainer to obtain cell suspensions.

#### Cell cultures

CD4^+^CD25 − CD62L^+^ naïve T cells used for in vitro experiments were sorted from spleens and lymph nodes of mice by magnetic cell sorting according to manufacturer’s protocol. Naïve T cells were cultured in plates coated with anti-mouse-CD3 (1.5 μg/mL) and soluble anti-mouse-CD28 (1.5 μg/mL), with or without CPT-11 (5, 10, 15, or 20 μg/mL), mouse IL-12 (10 ng/mL), TGF-β (2 ng/mL), IL-6 (50 ng/mL), 2-DG (5 μM). Three days later, cells were harvested for FCM. Samples were allocated randomly to treatment groups and ctrl groups in an arbitrary order; However, we didn’t use formal randomization techniques. Sample size was determined by previous similar studies [76, 77], and to ensure adequate data values to conduct standard statistical tests. Due to the investigators needs to design the treatment groups to conduct the experiments, this part did not blind to the group allocation.

#### Metabolic analyses

Seahorse XFe96 Analyzers was used to measure the extracellular acidification rate (ECAR) and oxygen consumption rate (OCR) of CD4^+^ T cells treated with CPT-11 (10 μg/mL) or PBS for 24 h. 2 × 10^5^ cultured T cells, resuspended in XF RPMI medium supplemented with 10 mM glucose, 1 mM pyruvate and 2 mM L-glutamine, were added into Seahorse XF96 cell culture microplates (coated with 22.4 μg/mL Corning® Cell-Tak™ Cell and Tissue Adhesive). In the absence of CO_2_, cells in microplates were incubated with shaking at 37 °C for approximately 30 min, and, subsequently, the Seahorse XF Cell Mito Stress Test program was run. To measure basal glycolysis in T cells treated with CPT-11 or PBS, the indicated cells were harvested after 24 h. Next, the cells were resuspended in the aforementioned XF RPMI medium and incubated with CPT-11 or PBS, and basal ECAR was measured.

#### FCM analyses

Dead and antigen presenting cells were stained or blocked by incubating the cells with zombie yellow and anti-mouse CD16/32 antibodies for 15 min at room temperature in the dark. Cell surface markers were stained by incubating cells with antibody solutions for 20 min at 4 °C in the dark. Cells were then fixed with Foxp3/Transcription Factor Staining Buffer Set or Cytofix/Cytoperm Fixation/Permeabilization Solution Kit according to the manufacturer’s protocol. Intranuclear and intracellular staining solutions were applied with buffer from the aforementioned kit, and, subsequently, single cell suspensions were stained with this solution for 40 min at 4 °C in the dark. For intracellular cytokine staining, cells were stimulated with PMA (10 ng/mL), ionomycin (250 ng/mL) and Golgi-Plug (1:1,000 dilution; BD Pharmingen) at 37 °C for 4 h, For cell apoptosis staining, cells were stained with Annexin V and 7-AAD solution diluted with Annexin Binding Buffer for 30 min at room temperature. Stained cells were analyzed using an LSRFortessa flow cytometer (BD Biosciences) and the data were analyzed using FlowJo software.

#### RNA-sequence

RNA-seq library preparation and sequencing for naïve T cells treated with different concentrations of CPT-11 were performed by Seqhealth Technology Co., LTD (Wuhan, China) on Illumina Novaseq 6000 machine. Raw sequencing data were first processed by Trimmomatic (version 0.36) to discard low-quality reads. Clean data were mapped to the mm10 mouse genome assembly by STRA software (version 2.5.3a). Differentially expressed genes were identified by DEseq2 (*p* < 0.05 and |Log2FoldChange | ≥1). Volcano plot and heat map showed differentially expressed genes related to T cell proliferation and apoptosis.

#### Quantitative RT-PCR

Total RNA was extracted from cultured cells using a RNeasy mini kit (Qiagen), and cDNA was obtained using a cDNA reverse transcription kit (Applied Biosystems). Quantitative real-time PCR was performed using SYBR Green Real-Time PCR Master Mix (Toyobo). The results were normalized to the expression of HPRT mRNA.

**Table:** 

Oligonucleotides	
Hprt-forward 5’	TCAGTCAACGGGGGACATAAA
Hprt-reverse 5’	GGGGCTGTACTGCTTAACCAG
Hk1-forward 5’	CGGAATGGGGAGCCTTTGG
Hk1-reverse 5’	GCCTTCCTTATCCGTTTCAATGG
Hk2-forward 5’	TGATCGCCTGCTTATTCACGG
Hk2-reverse 5’	AACCGCCTAGAAATCTCCAGA
Hk3-forward 5’	CAGGGGACCTACAGGATTGAT
Hk3-reverse 5’	GAGCATCTTCGTCATAGAAGGAG
Hk4-forward 5’	TGAGCCGGATGCAGAAGGA
Hk4-reverse 5	GCAACATCTTTACACTGGCCT
Pfk-P-forward 5’	GAAACATGAGGCGTTCTGTGT
Pfk-P-reverse 5’	CCCGGCACATTGTTGGAGA
Pfk-L-forward 5’	GGAGGCGAGAACATCAAGCC
Pfk-L-reverse 5’	CGGCCTTCCCTCGTAGTGA
Pfk-M-forward 5’	TGTGGTCCGAGTTGGTATCTT
Pfk-M-reverse 5’	GCACTTCCAATCACTGTGCC
Pk-M-forward 5’	GCCGCCTGGACATTGACTC
Pk-M-reverse 5’	CCATGAGAGAAATTCAGCCGAG
Aldo-a-forward 5’	CGTGTGAATCCCTGCATTGG
Aldo-a-reverse 5’	CAGCCCCTGGGTAGTTGTC
Tpi-1-forward 5’	CCAGGAAGTTCTTCGTTGGGG
Tpi-1-reverse 5’	CAAAGTCGATGTAAGCGGTGG
Tnf-forward 5’	CCCTCACACTCAGATCATCTTCT
Tnf-reverse 5’	GCTACGACGTGGGCTACAG
Zbtb32-forward 5’	GGTACAGTTAGCGGCTAGACT
Zbtb32-reverse 5’	GGAAGGGCTTATGTCTTCAACC
Cdkn1a-forward 5’	CCTGGTGATGTCCGACCTG
Cdkn1a-reverse 5’	CCATGAGCGCATCGCAATC
Tigit-forward 5’	GAATGGAACCTGAGGAGTCTCT
Tigit-reverse 5’	AGCAATGAAGCTCTCTAGGCT
Gpr55-forward 5’	CACTAAGGGCTGGGTACAAAAG
Gpr55-reverse 5’	GCGGTTCCTCACCAGATACTG
Perp-forward 5’	ATCGCCTTCGACATCATCGC
Perp-reverse 5’	CCCCATGCGTACTCCATGAG
Phlda1-forward 5’	GGGCTACTGCTCATACCGC
Phlda1-reverse 5’	AAAAGTGCAATTCCTTCAGCTTG
Prkca-forward 5’	GTTTACCCGGCCAACGACT
Prkca-reverse 5’	GGGCGATGAATTTGTGGTCTT
Pros1-forward 5’	CGCTTTCGGGTGCTACTGG
Pros1-reverse 5’	CACTCTCGTTCAAGGTTGCC
Skap1-forward 5’	AGGACGAGGGAATAGAAGACATC
Skap1-reverse 5’	TTCTTGGAATCTTTTCGCAGGT
Snd1-forward 5’	TCTGGGTGCGCCATAATTGTC
Snd1-reverse 5’	TCAGCTTCTTGCGAAGGAACT
Themis-forward 5’	AGTCACCATGTAGACAGACCC
Themis-reverse 5’	GTGGCCCATGCTTGCTCTT
Txnip-forward 5’	TCTTTTGAGGTGGTCTTCAACG
Txnip-reverse 5’	GCTTTGACTCGGGTAACTTCACA
Zbtb20-forward 5’	GCGAGCCCAAAGGTGAAAG
Zbtb20-reverse 5’	GCTGTAGGACGCCCTTATCG
Dock2-forward 5’	GCATCTCACGCTACAGATTGG
Dock2-reverse 5’	GGAAAATGCCCTGTGACAGTT

### Quantification and statistical analysis

All data were analyzed using GraphPad Prism 9 software. An unpaired two-tailed Student’s t-test was used to compare variables between two groups; one-way ANOVA (with Tukey’s multiple-comparison post-tests) was used to compare variables between more than two groups. All *P*-values < 0.05 were considered to be statistically significant (*p < 0.05, **p < 0.01, ***p < 0.001, ****p < 0.0001, NS- not significant).

### Supplementary information

Supplementary Figure 1-8

## Data Availability

RNA-seq data has been uploaded on public database and can be found in the GSA database (CRA013136).

## References

[1] Pillai S (2013). Rethinking mechanisms of autoimmune pathogenesis. J Autoimmun.

[2] Raje N, Dinakar C (2015). Overview of Immunodeficiency Disorders. Immunol Allerg Clin North America.

[3] Sakaguchi S, Sakaguchi N, Asano M, Itoh M, Toda M (1995). Immunologic self-tolerance maintained by activated T cells expressing IL-2 receptor alpha-chains (CD25). Breakdown of a single mechanism of self-tolerance causes various autoimmune diseases. J Immunol. (Baltimore, Md.: 1950).

[4] Ahmadi M, Gharibi T, Dolati S, Rostamzadeh D, Aslani S, Baradaran B (2017). Epigenetic modifications and epigenetic based medication implementations of autoimmune diseases. Biomed Pharmacother. = Biomedecine & pharmacotherapie.

[5] Davidson A, Diamond B (2001). Autoimmune diseases. New Engl J Med.

[6] Oo YH, Hubscher SG, Adams DH (2010). Autoimmune hepatitis: new paradigms in the pathogenesis, diagnosis, and management. Hepatol Int.

[7] Wang H, Zhang D, Han Q, Zhao X, Zeng X, Xu Y (2016). Role of distinct CD4(+) T helper subset in pathogenesis of oral lichen planus. J Oral Pathol Med.

[8] Harkiolaki M, Holmes SL, Svendsen P, Gregersen JW, Jensen LT, McMahon R (2009). T cell-mediated autoimmune disease due to low-affinity crossreactivity to common microbial peptides. Immunity.

[9] Hahn RZ, Antunes MV, Verza SG, Perassolo MS, Suyenaga ES, Schwartsmann G (2019). Pharmacokinetic and Pharmacogenetic Markers of Irinotecan Toxicity. Curr Med Chem.

[10] Langer CJ (2001). The emerging world role of irinotecan in lung cancer. Oncology (Williston Park, N.Y.).

[11] Makiyama A, Arimizu K, Hirano G, Makiyama C, Matsushita Y, Shirakawa T (2018). Irinotecan monotherapy as third-line or later treatment in advanced gastric cancer. Gastric Cancer: Off J Int Gastric Cancer Asso Japanese Gastric Cancer Asso.

[12] Gershenson DM (2002). Irinotecan in epithelial ovarian cancer. Oncology (Williston Park, N.Y.).

[13] Verschraegen CF (2002). Irinotecan for the treatment of cervical cancer. Oncology (Williston Park, N.Y.).

[14] Fuchs C, Mitchell EP, Hoff PM (2006). Irinotecan in the treatment of colorectal cancer. Cancer Treat Rev.

[15] Xu Y, Li Q, Ma HY, Sun T, Xiang RL, Di F (2020). Therapeutic effect and side effects of Bevacizumab combined with Irinotecan in the treatment of paediatric intracranial tumours: Meta-analysis and Systematic Review. J Clin Pharmacy Therapeutics.

[16] Kurita A, Kado S, Kaneda N, Onoue M, Hashimoto S, Yokokura T (2003). Alleviation of side effects induced by irinotecan hydrochloride (CPT-11) in rats by intravenous infusion. Cancer chemother Pharmacol.

[17] Scholzen T, Gerdes J (2000). The Ki-67 protein: from the known and the unknown. J Cell Physiol.

[18] Bernard M, Yang B, Migneault F, Turgeon J, Dieudé M, Olivier MA (2020). Autophagy drives fibroblast senescence through MTORC2 regulation. Autophagy.

[19] Brenner D, Blaser H, Mak TW (2015). Regulation of tumour necrosis factor signalling: live or let die. Nat Rev Immunol.

[20] Oberg HH, Sipos B, Kalthoff H, Janssen O, Kabelitz D (2004). Regulation of T-cell death-associated gene 51 (TDAG51) expression in human T-cells. Cell Death Differ.

[21] Zhou Y, Leng X, He Y, Li Y, Liu Y, Liu Y (2018). Loss of Perp in T Cells Promotes Resistance to Apoptosis of T Helper 17 Cells and Exacerbates the Development of Experimental Autoimmune Encephalomyelitis in Mice. Front Immunol.

[22] Chauvin, JM & Zarour, HM TIGIT in cancer immunotherapy. J Immunother Cancer 2020 8. 10.1136/jitc-2020-000957 10.1136/jitc-2020-000957 PMC7477968 32900861

[23] Coley WD, Zhao Y, Benck CJ, Liu Y, Hotta-Iwamura C, Rahman MJ (2018). Loss of Zbtb32 in NOD mice does not significantly alter T cell responses. F1000Res.

[24] Yue C, Gao S, Li S, Xing Z, Qian H, Hu Y (2022). TIGIT as a Promising Therapeutic Target in Autoimmune Diseases. Front Immunol.

[25] Zhai K, Shi XY, Yi FS, Huang ZY, Wu XZ, Dong SF (2020). IL-10 promotes malignant pleural effusion by regulating T(H) 1 response via an miR-7116-5p/GPR55/ERK pathway in mice. Eur J Immunol.

[26] Alosaimi MF, Shendi H, Beano A, Stafstrom K, El Hawary R, Meshaal S (2019). T-cell mitochondrial dysfunction and lymphopenia in DOCK2-deficient patients. J Allerg Clin Immunol.

[27] Krzyzanowska AK, Haynes Ii RAH, Kovalovsky D, Lin HC, Osorio L, Edelblum KL (2022). Zbtb20 identifies and controls a thymus-derived population of regulatory T cells that play a role in intestinal homeostasis. Sci Immunol.

[28] Liu Y, Cong Y, Niu Y, Yuan Y, Tan F, Lai Q (2022). Themis is indispensable for IL-2 and IL-15 signaling in T cells. Sci Signal.

[29] Mehta M, Brzostek J, Chen EW, Tung DWH, Chen S, Sankaran S (2018). Themis-associated phosphatase activity controls signaling in T cell development. Proc Natl Acad Sci USA.

[30] Raab M, Strebhardt K, Rudd CE (2019). Immune adaptor SKAP1 acts a scaffold for Polo-like kinase 1 (PLK1) for the optimal cell cycling of T-cells. Sci Rep.

[31] Wang J, Wu N, Feng X, Liang Y, Huang M, Li W (2022). PROS1 shapes the immune-suppressive tumor microenvironment and predicts poor prognosis in glioma. Front Immunol.

[32] Zhao L, Hsiao T, Stonesifer C, Daniels J, Garcia-Saleem TJ, Choi J (2022). The Robust Tumoricidal Effects of Combined BET/HDAC Inhibition in Cutaneous T-Cell Lymphoma Can Be Reproduced by ΔNp73 Depletion. J Investig Dermatol.

[33] Huang J, Li Z, Hu Y, Li Z, Xie Y, Huang H (2022). Melatonin, an endogenous hormone, modulates Th17 cells via the reactive-oxygen species/TXNIP/HIF-1α axis to alleviate autoimmune uveitis. J Neuroinflamm.

[34] Wang X, Zhang C, Wang S, Rashu R, Thomas R, Yang J (2021). SND1 promotes Th1/17 immunity against chlamydial lung infection through enhancing dendritic cell function. PLoS Pathogens.

[35] Frauwirth KA, Riley JL, Harris MH, Parry RV, Rathmell JC, Plas DR (2002). The CD28 signaling pathway regulates glucose metabolism. Immunity.

[36] Gerriets VA, Kishton RJ, Nichols AG, Macintyre AN, Inoue M, Ilkayeva O (2015). Metabolic programming and PDHK1 control CD4+ T cell subsets and inflammation. J Clin Investig.

[37] Marjanovic S, Eriksson I, Nelson BD (1990). Expression of a new set of glycolytic isozymes in activated human peripheral lymphocytes. Biochimica et biophysica acta.

[38] Zuo J, Tang J, Lu M, Zhou Z, Li Y, Tian H (2021). Glycolysis Rate-Limiting Enzymes: Novel Potential Regulators of Rheumatoid Arthritis Pathogenesis. Front Immunol.

[39] Wang R, Dillon CP, Shi LZ, Milasta S, Carter R, Finkelstein D (2011). The transcription factor Myc controls metabolic reprogramming upon T lymphocyte activation. Immunity.

[40] van der Fits L, Mourits S, Voerman JS, Kant M, Boon L, Laman JD (2009). Imiquimod-induced psoriasis-like skin inflammation in mice is mediated via the IL-23/IL-17 axis. J Immunol (Baltimore, Md.: 1950).

[41] Vredenburgh JJ, Desjardins A, Reardon DA, Friedman HS (2009). Experience with irinotecan for the treatment of malignant glioma. Neuro Oncol.

[42] Friedman HS, Keir ST, Houghton PJ (2003). The emerging role of irinotecan (CPT-11) in the treatment of malignant glioma in brain tumors. Cancer.

[43] Cua DJ, Sherlock J, Chen Y, Murphy CA, Joyce B, Seymour B (2003). Interleukin-23 rather than interleukin-12 is the critical cytokine for autoimmune inflammation of the brain. Nature.

[44] Rees F, Doherty M, Grainge M, Davenport G, Lanyon P, Zhang W (2016). The incidence and prevalence of systemic lupus erythematosus in the UK, 1999-2012. Ann Rheum Dis.

[45] Zhang D, Jin W, Wu R, Li J, Park SA, Tu E (2019). High Glucose Intake Exacerbates Autoimmunity through Reactive-Oxygen-Species-Mediated TGF-beta Cytokine Activation. Immunity.

[46] Rose NR (2016). Prediction and Prevention of Autoimmune Disease in the 21st Century: A Review and Preview. Am J Epidemiol.

[47] Sun G, Hou Y, Gong W, Liu S, Li J, Yuan Y (2018). Adoptive Induced Antigen-Specific Treg Cells Reverse Inflammation in Collagen-Induced Arthritis Mouse Model. Inflammation.

[48] Zhan Q, Zhang J, Lin Y, Chen W, Fan X, Zhang D (2023). Pathogenesis and treatment of Sjogren’s syndrome: Review and update. Front Immunol.

[49] Zhang D, Tu E, Kasagi S, Zanvit P, Chen Q, Chen W (2015). Manipulating regulatory T cells: a promising strategy to treat autoimmunity. Immunotherapy.

[50] Chapman TM, Keating GM (2003). Basiliximab: a review of its use as induction therapy in renal transplantation. Drugs.

[51] Chukwu CA, Spiers HVM, Middleton R, Kalra PA, Asderakis A, Rao A (2022). Alemtuzumab in renal transplantation. Reviews of literature and usage in the United Kingdom. Transpl Rev. (Orlando, Fla.).

[52] Evan JR, Bozkurt SB, Thomas NC, Bagnato F (2018). Alemtuzumab for the treatment of multiple sclerosis. Exp Opin Biol Ther.

[53] Wu Q, Wang X, Nepovimova E, Wang Y, Yang H, Kuca K (2018). Mechanism of cyclosporine A nephrotoxicity: Oxidative stress, autophagy, and signalings. Food Chem Toxicol: Int J Published Brit Indus Biol Res Assoc.

[54] Farouk SS, Rein JL (2020). The Many Faces of Calcineurin Inhibitor Toxicity-What the FK?. Adv Chronic Kidney Dis.

[55] Wilk A, Szypulska-Koziarska D, Kędzierska-Kapuza K, Kolasa-Wołosiuk A, Misiakiewicz-Has K, Ciechanowski K (2018). Effect of long-term immunosuppressive therapy on native rat liver morphology and hepatocyte- apoptosis. Transpl Immunol.

[56] Trager J, Ward MM (2001). Mortality and causes of death in systemic lupus erythematosus. Curr Opin Rheumatol.

[57] Qi WX, Huang YJ, Yao Y, Shen Z, Min DL (2013). Incidence and risk of treatment-related mortality with mTOR inhibitors everolimus and temsirolimus in cancer patients: a meta-analysis. PloS one.

[58] Bereshchenko O, Bruscoli S, Riccardi C (2018). Glucocorticoids, Sex Hormones, and Immunity. Front Immunol.

[59] Wong SH, Gao Q, Tsoi KK, Wu WK, Tam LS, Lee N (2016). Effect of immunosuppressive therapy on interferon gamma release assay for latent tuberculosis screening in patients with autoimmune diseases: a systematic review and meta-analysis. Thorax.

[60] Zhang D, Wang J, Li Z, Zhou M, Chen Q, Zeng X (2015). The Activation of NF-kappaB in Infiltrated Mononuclear Cells Negatively Correlates with Treg Cell Frequency in Oral Lichen Planus. Inflammation.

[61] Kornberg MD, Bhargava P, Kim PM, Putluri V, Snowman AM, Putluri N (2018). Dimethyl fumarate targets GAPDH and aerobic glycolysis to modulate immunity. Science (New York, N.Y.).

[62] Chávez MD, Tse HM (2021). Targeting Mitochondrial-Derived Reactive Oxygen Species in T Cell-Mediated Autoimmune Diseases. Front Immunol.

[63] Serra P, Santamaria P (2019). Antigen-specific therapeutic approaches for autoimmunity. Nat Biotechnol.

[64] Fang ZZ, Zhang D, Cao YF, Xie C, Lu D, Sun DX (2016). Irinotecan (CPT-11)-induced elevation of bile acids potentiates suppression of IL-10 expression. Toxicol Appl Pharmacol.

[65] Han MN, Finkelstein DI, McQuade RM, Diwakarla S. Gastrointestinal Dysfunction in Parkinson’s Disease: Current and Potential Therapeutics. J Personal Med. 2022;12. 10.3390/jpm12020144 10.3390/jpm12020144 PMC8875119 35207632

[66] Fourie Zirkelbach J, Shah M, Vallejo J, Cheng J, Ayyoub A, Liu J (2022). Improving Dose-Optimization Processes Used in Oncology Drug Development to Minimize Toxicity and Maximize Benefit to Patients. J Clin Oncol: Off J Am Soc Clin Oncol.

[67] Wu Q, Miao X, Zhang J, Xiang L, Li X, Bao X (2021). Astrocytic YAP protects the optic nerve and retina in an experimental autoimmune encephalomyelitis model through TGF-β signaling. Theranostics.

[68] Thomann AS, McQuade CA, Pinjušić K, Kolz A, Schmitz R, Kitamura D (2023). A B cell-driven EAE mouse model reveals the impact of B cell-derived cytokines on CNS autoimmunity. Proc Natl Acad Sci USA.

[69] Hou Y, Zhu L, Tian H, Sun HX, Wang R, Zhang L (2018). IL-23-induced macrophage polarization and its pathological roles in mice with imiquimod-induced psoriasis. Protein Cell.

[70] Yin M, Guo Y, Hu R, Cai WL, Li Y, Pei S (2020). Potent BRD4 inhibitor suppresses cancer cell-macrophage interaction. Nat Commun.

[71] Wang Z, Sun Y, Lou F, Bai J, Zhou H, Cai X (2022). Targeting the transcription factor HES1 by L-menthol restores protein phosphatase 6 in keratinocytes in models of psoriasis. Nat Commun.

[72] Konkel JE, Zhang D, Zanvit P, Chia C, Zangarle-Murray T, Jin W (2017). Transforming Growth Factor-β Signaling in Regulatory T Cells Controls T Helper-17 Cells and Tissue-Specific Immune Responses. Immunity.

[73] Yong L, Yu Y, Li B, Ge H, Zhen Q, Mao Y (2022). Calcium/calmodulin-dependent protein kinase IV promotes imiquimod-induced psoriatic inflammation via macrophages and keratinocytes in mice. Nat Commun.

[74] Zanvit P, Konkel JE, Jiao X, Kasagi S, Zhang D, Wu R (2015). Antibiotics in neonatal life increase murine susceptibility to experimental psoriasis. Nat Commun.

[75] Perruche S, Zhang P, Liu Y, Saas P, Bluestone JA, Chen W (2008). CD3-specific antibody-induced immune tolerance involves transforming growth factor-beta from phagocytes digesting apoptotic T cells. Nat Med.

[76] Zhang D, Chia C, Jiao X, Jin W, Kasagi S, Wu R (2017). D-mannose induces regulatory T cells and suppresses immunopathology. Nat Med.

[77] Zhang D, Jin W, Wu R, Li J, Park SA, Tu E (2019). High Glucose Intake Exacerbates Autoimmunity through Reactive-Oxygen-Species-Mediated TGF-β Cytokine Activation. Immunity.

