# Supplementary material for: CPT-11 mitigates autoimmune diseases by suppressing effector T cells without affecting long-term anti-tumor immunity
Source: Cell Death Discov. 2024 May 4;10:218. doi: 10.1038/s41420-024-01983-8 (PMC11069576; doi:10.1038/s41420-024-01983-8)
Supplement: Supplementary file 1 — Supplementary Figure 1-8 [file 41420_2024_1983_MOESM1_ESM.pdf]

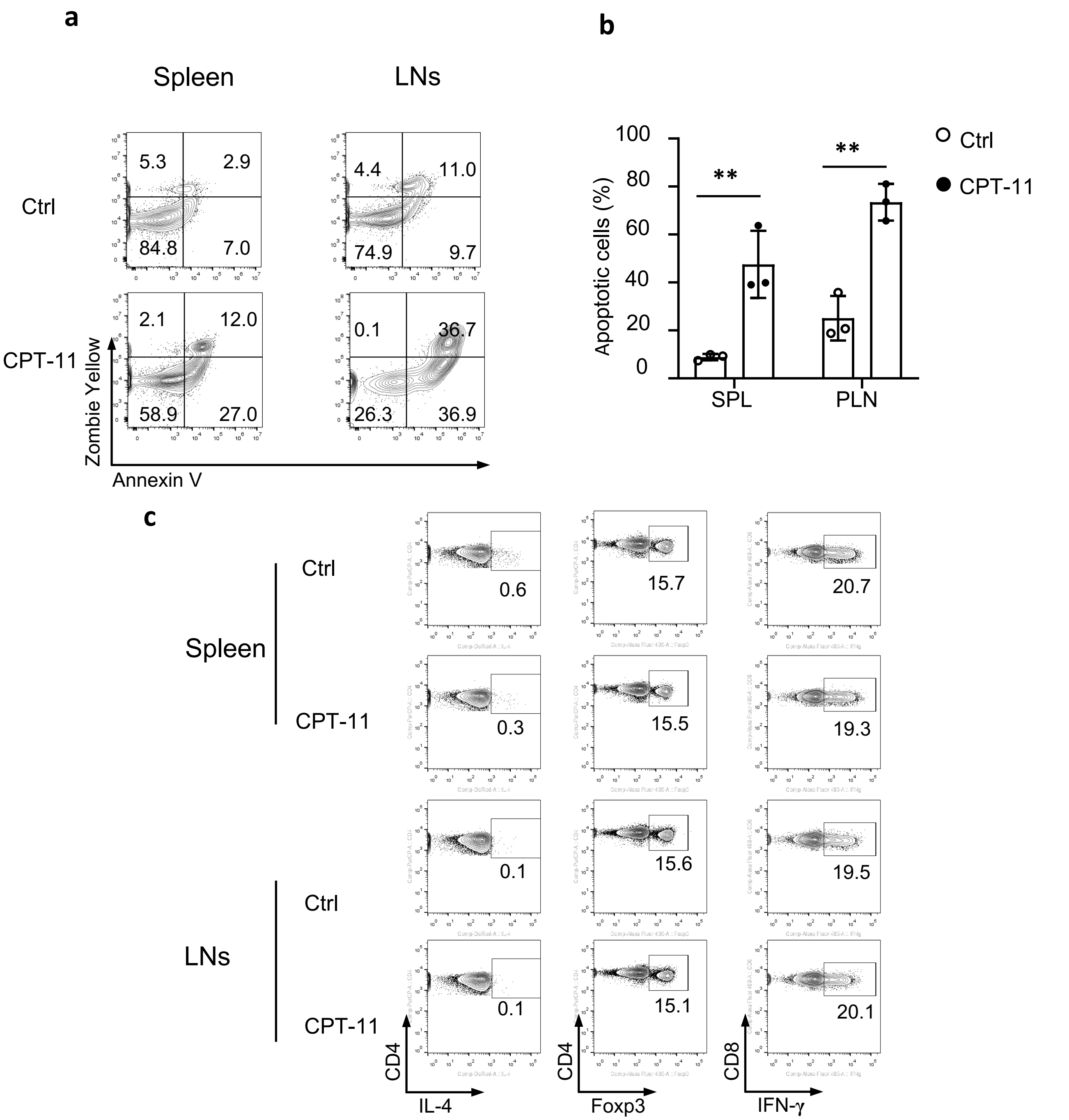

Supplementary Fig. 1. CPT-11 reduces immune cell numbers and suppresses cytokine production of T cells, related to Fig. 1. C57BL/6 mice were treated with CPT-11 and the immune responses were determined by flow cytometry (FCM). (a,b) Representative FACS plots and bar graph showing frequencies of apoptotic cells in indicated mice. (c) Representative FACS plots of indicated groups. Data are representative of two independent experiments. Summary data are presented as mean ± s.d. $^{**}p<0.01$; by unpaired two-tailed Student's t-tests.

Fig. S1

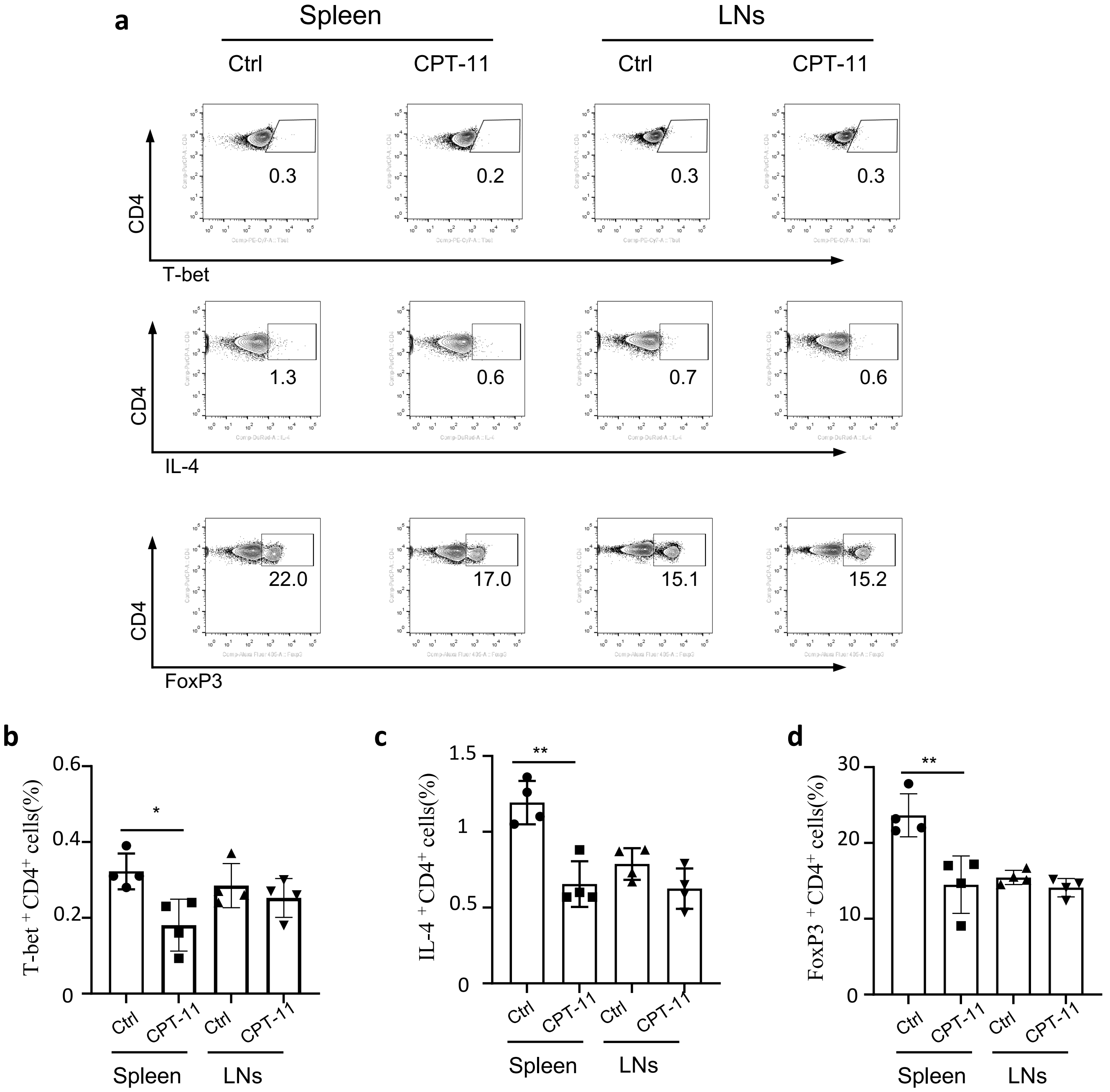

Supplementary Fig. 2. CPT-11 restrains systemic inflammation under CFA immune challenge, related to Fig. 2.

C57BL/6 mice were challenged with CFA (subcutaneous injection) and treated with CPT-11 or PBS, and the immune responses in spleen and LNs were determined by FCM. (a) Representative FACS plots. (b-d) Bar graphs showing frequencies of T-bet+ CD4+ Th1 cells, IL-4+ CD4+ Th2 cells and FoxP3+ CD4+ Treg cells in indicated mice. Data are representative of two independent experiments. Summary data are presented as mean ± s.d. *p<0.05, **p<0.01; by unpaired two-tailed Student's t-tests.

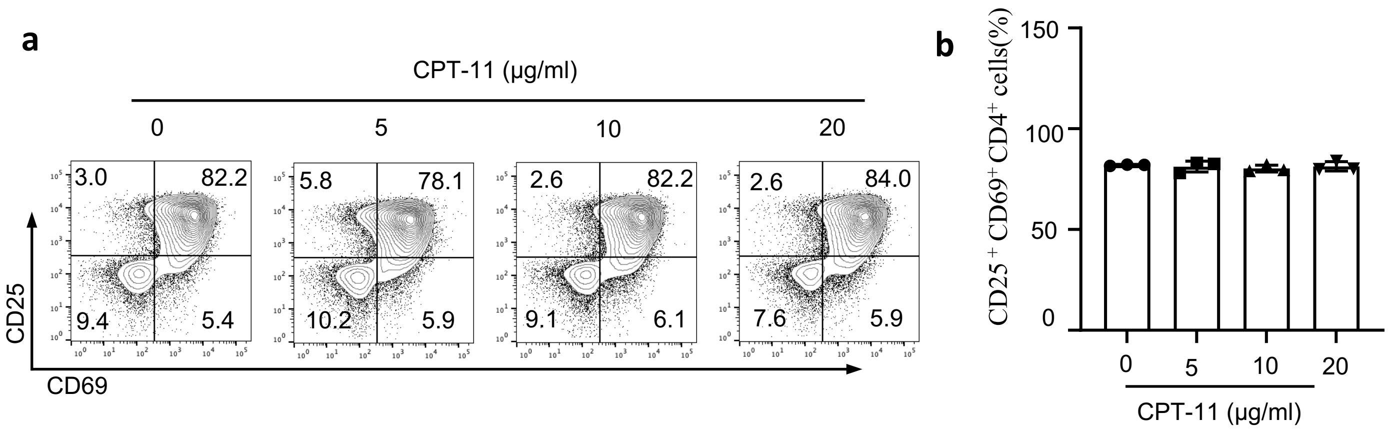

Supplementary Fig. 3. CPT-11 does not suppresses T cell activation *in vitro*, related to Fig. 3.

CD4$^+$CD25$^-$CD62L^{high} (naive) T cells isolated from spleen and LNs of C57BL/6 mice were cultured with anti-mouse-CD3 and anti-mouse-CD28 for 24 hrs *in vitro* (n=3). (a-b) Representative FACS plots (a) and bar graph (b) showing activated T cell frequencies in T cells cultured for 24 hrs. Data are representative of three independent experiments (a) or are pooled from three independent experiments (b). Summary data are presented as mean ± s.d.

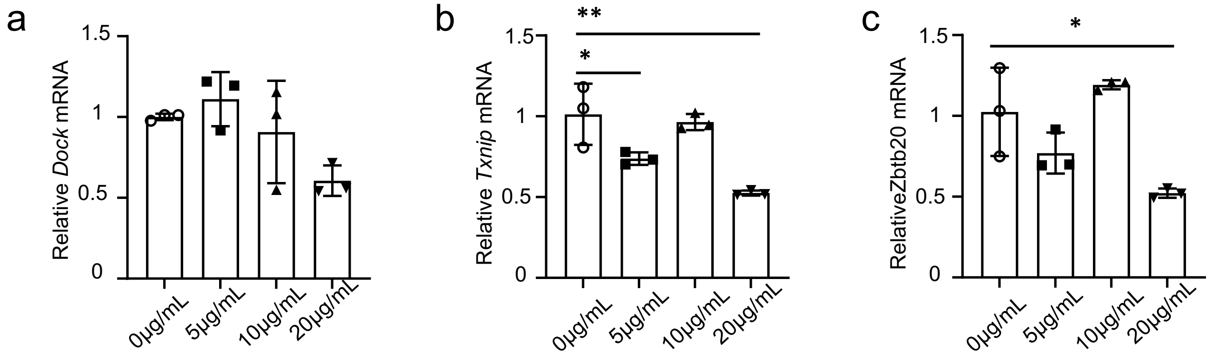

Supplementary Fig. 4. CPT-11 suppresses CD4⁺ T cell glycolysis, related to Fig. 4. Naive T cells isolated from C57BL/6 mice were cultured for 1 d with anti-CD3 and anti-CD28, with or without CPT-11. (a-c) Dock, Txnip and Zbtb20 mRNA expression in T cells cultured for 1 d with different concentrations (0 μg/mL, 5 μg/mL, 10 μg/mL, and 20 μg/mL) of CPT-11. Data are pooled from three independent experiments. Summary data are presented as mean ± s.d. *p <0.05, **p <0.01; by one-way analysis of variance (ANOVA) with Tukey's post hoc test.

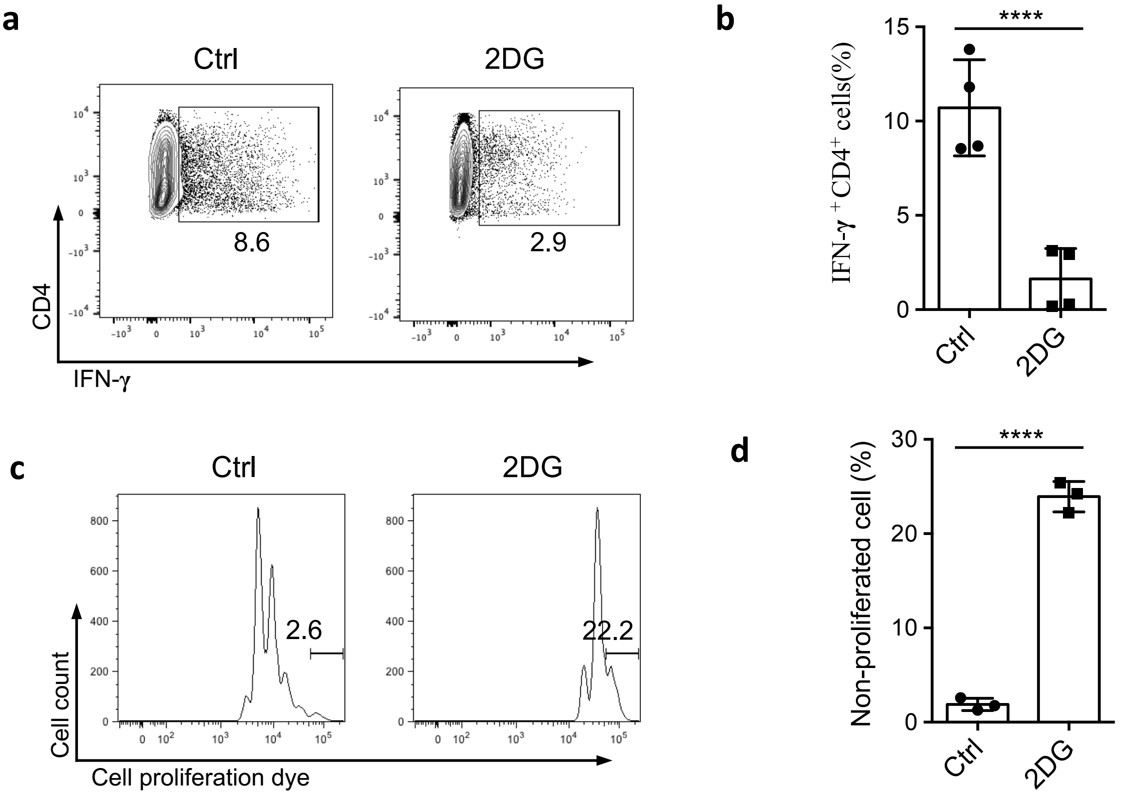

Supplementary Fig. 5. CPT-11 suppresses CD4+ T cell proliferation and cytokine production by inhibiting glycolysis, related to Fig. 5.

Naive T cells isolated from C57BL/6 mice were cultured for 3 d with anti-CD3 and anti-CD28, with or without 2-DG. (a-b) Representative FACS plots (a) and bar graph showing frequencies of IFN-γ+ CD4+ Th1 cells (b). (c-d) Representative FACS plots and bar graph showing frequencies of non-proliferated cells among CD4+ T cells. Data are representative of three (c) or four (a) independent experiments or are pooled from three (d) or four (b) independent experiments. Summary data are presented as mean ± s.d. ****$p<0.0001$; by unpaired two-tailed Student's t-tests.

Fig. S5

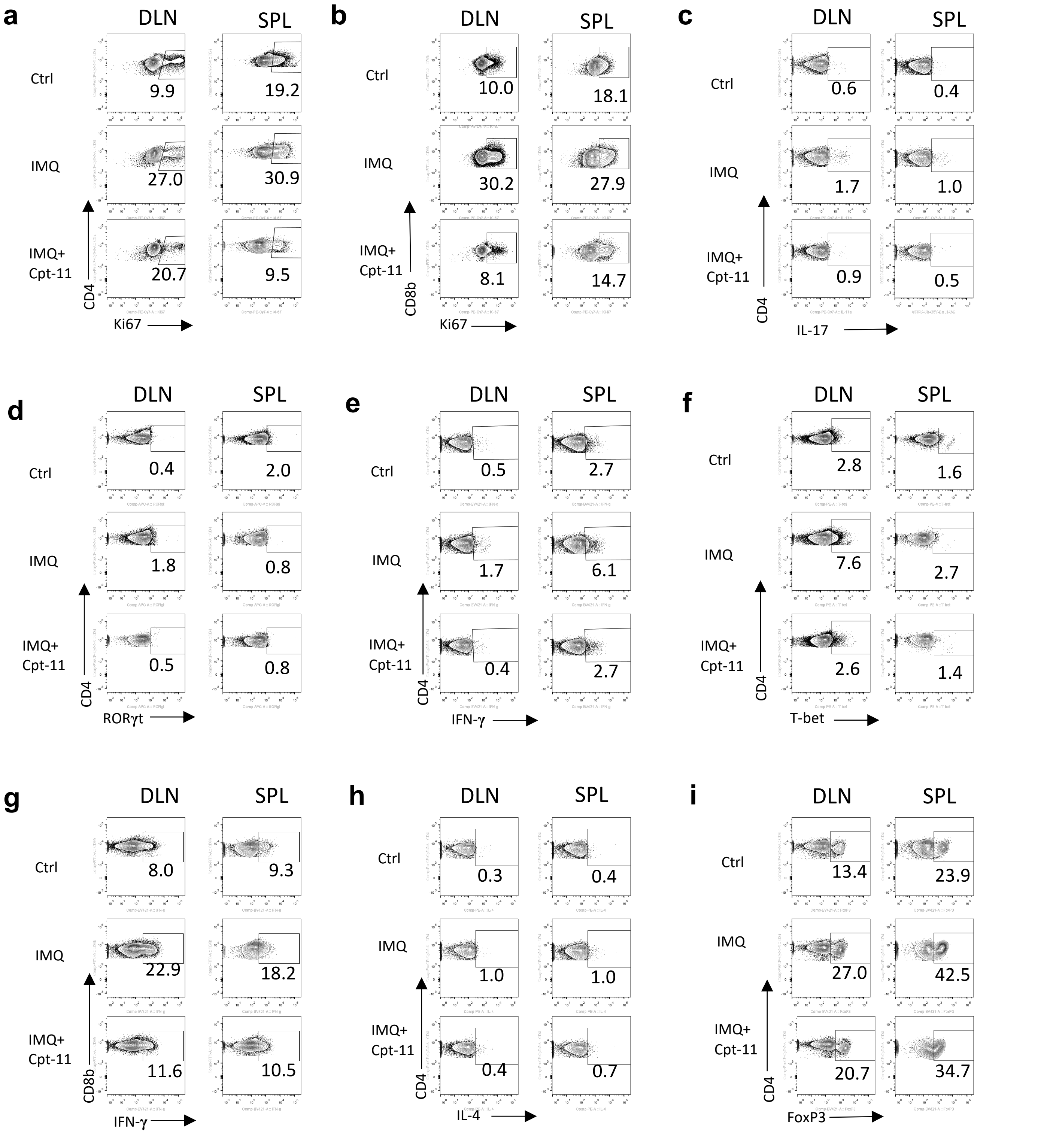

Supplementary Fig. 6. CPT-11 suppresses the progression of psoriasis, related to Fig. 6.

C57BL/6 mice were applied with IMQ cream on shaved 2.5 cm × 2.5 cm back skin daily for 7 consecutive days, and were injected with CPT-11 or PBS intraperitoneally once per day (n=12 mice per group). (a-i) Representative FACS plots in indicated groups. Data are representative of three independent experiments.

Fig.S6

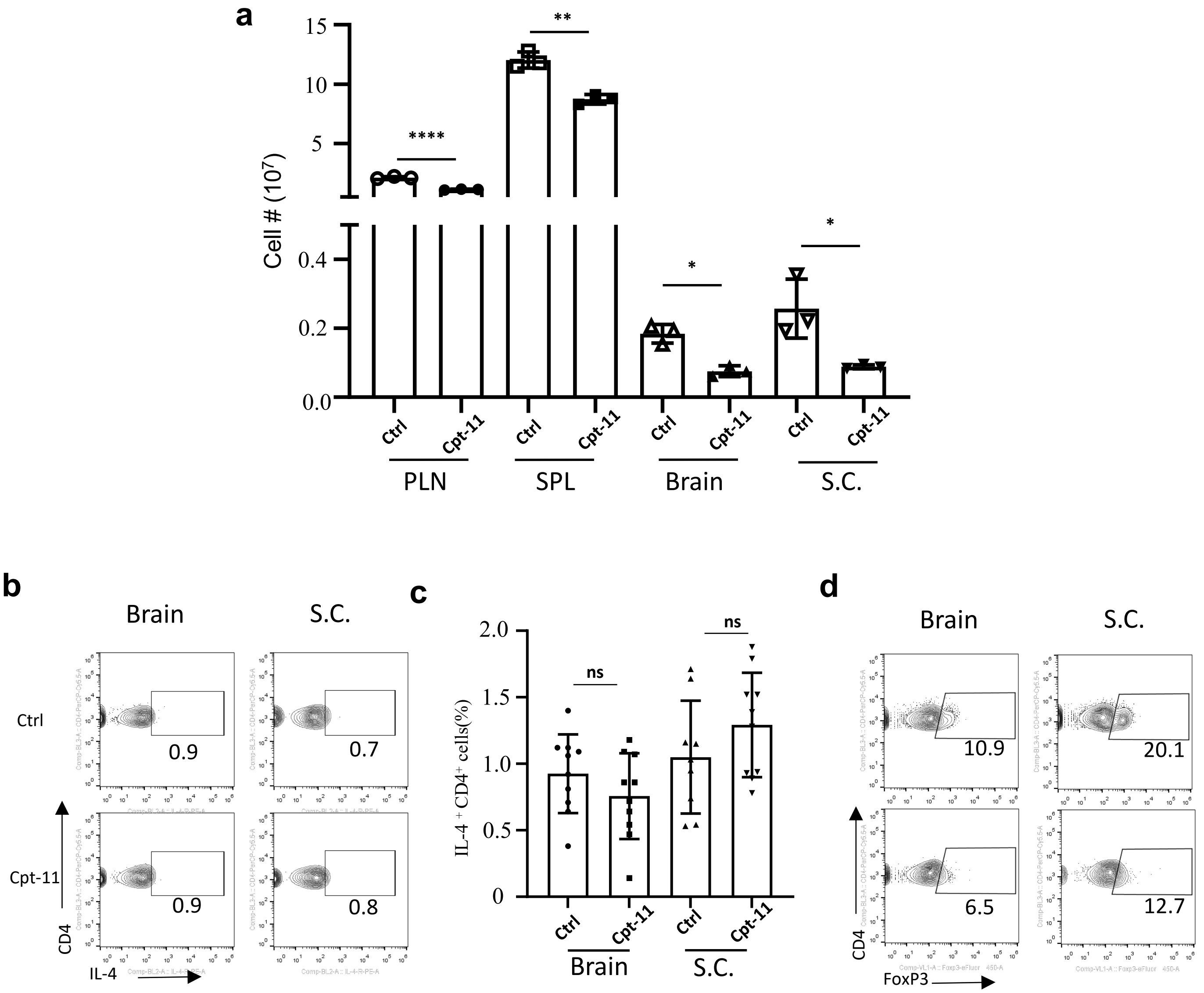

Supplementary Fig. 7. CPT-11 inhibits the development of EAE, related to Fig. 7. C57BL/6 mice were subcutaneously immunized with MOG peptide 35–55 emulsified in complete Freund's adjuvant to induce EAE, and treated with CPT-11 or PBS daily from day 9. (a) Total immune cell numbers of spleen, inguinal lymph nodes, brain and spinal cord in indicated groups. (b, d) Representative FACS plots and (c) bar graph showing frequencies of IL-4^+CD4^+ Th2 cells and Foxp3^+ Treg cells in brain and spinal cord. Data are representative of two independent experiments (a, b, d) or are pooled from two independent experiments (c). Summary data are presented as mean ± s.d. *$p<0.05$, **$p<0.01$, ****$p<0.0001$; by unpaired two-tailed Student's t-tests.

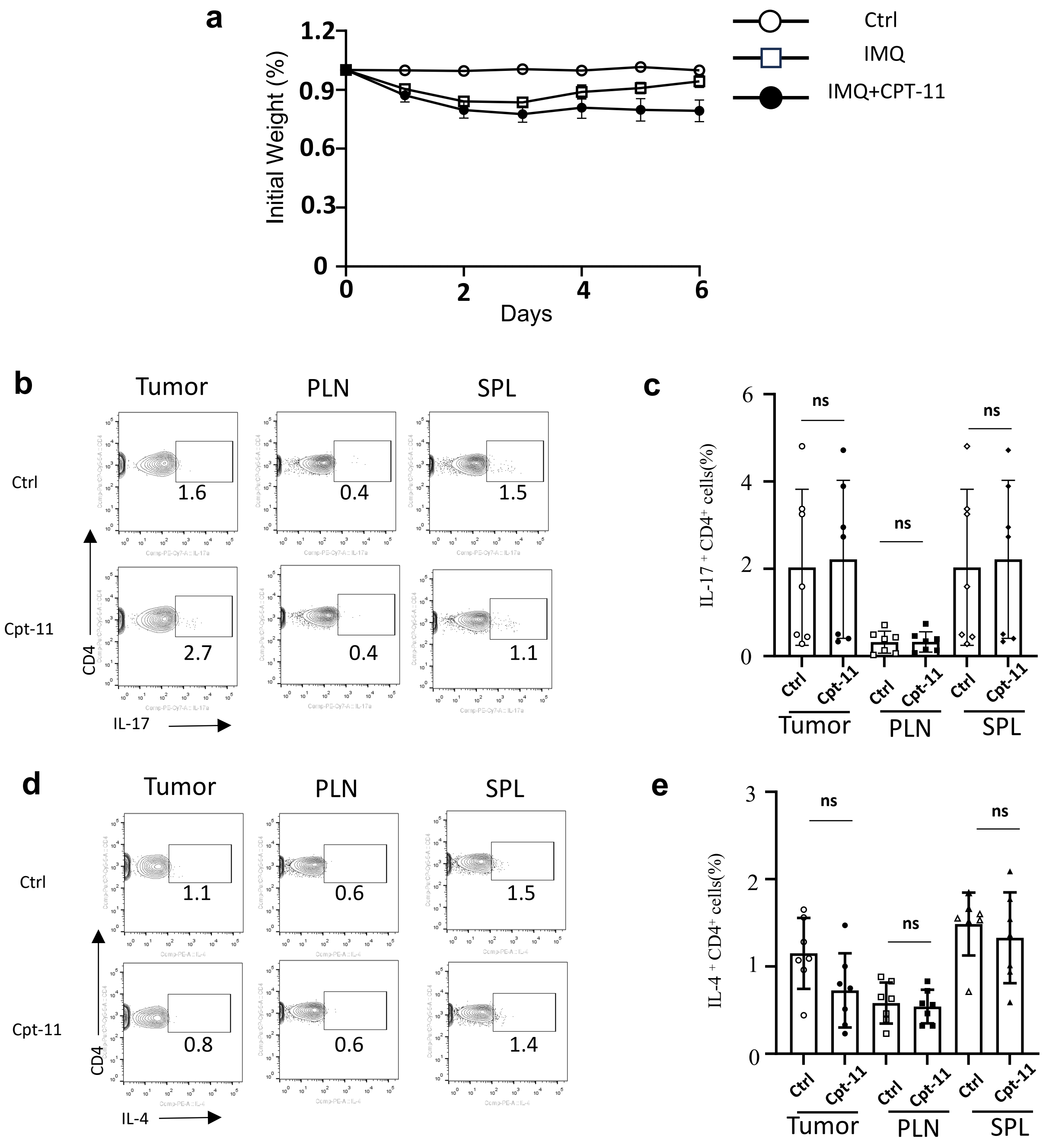

Supplementary Fig. 8. CPT-11 therapy of autoimmune diseases does not affect long-term anti-tumor immunity, related to Fig. 8.

(a) Body weight changes of mice treated with or without CPT-11 in psoriasis model. (b-e) C57BL/6 mice were administered IMQ cream on shaved 2.5 cm × 2.5 cm patches of back skin daily for 7 consecutive days and were injected with CPT-11 or PBS intraperitoneally once per day. Approximately 5 weeks after psoriasis induction and treatment, the mice were injected with B16 cells to establish a tumor-bearing model (n=7 mice per group). Representative FACS plots and bar graphs showing frequencies of IL-17+ CD4+ Th17 cells (b,c) and IL-4+ CD4+ Th2 cells (d,e). Data are representative of two independent experiments (a,b,d) or are pooled from two independent experiments (c,e).
